# Continuous ZnO nanoparticle exposure induces melanoma-like skin lesions in epidermal barrier dysfunction model mice through anti-apoptotic effects mediated by the oxidative stress–activated NF-κB pathway

**DOI:** 10.1186/s12951-022-01308-w

**Published:** 2022-03-05

**Authors:** Ping Wang, Guodong Hu, Wen Zhao, Juan Du, Menghan You, Mengying Xv, Hong Yang, Min Zhang, Fang Yan, Mianbo Huang, Xueer Wang, Lin Zhang, Yinghua Chen

**Affiliations:** 1 Department of Histology and Embryology, Guangdong Provincial Key Laboratory of Construction and Detection in Tissue Engineering, NMPA Key Laboratory for Safety Evaluation of Cosmetics, National Demonstration Center for Experimental Education, School of Basic Medical Sciences, Southern Medical University, Guangzhou, 510515 China; 2 Department of Respiratory and Critical Care Medicine, Nanfang Hospital, Southern Medical University, Guangzhou, 510515 China; 3 Department of Medical Imaging, Guangzhou Women and Children’s Medical Center, National Children’s Medical Center for South Central Region, Guangzhou, 510515 China; 4 Department of Inspection and Quarantine (Hygiene Detection Center), School of Public Health, Southern Medical University, Guangzhou, 510515 China; 5 Department of Respiratory and Critical Care Medicine, The People’s Hospital of Yangjiang, Yangjiang, 529500 China

**Keywords:** Zinc oxide nanoparticles, Epidermal barrier dysfunction skin, Melanocyte, Oxidative stress, Anti-apoptosis, NF-κB p65

## Abstract

**Background:**

Increasing interest in the hazardous properties of zinc oxide nanoparticles (ZnO NPs), commonly used as ultraviolet filters in sunscreen, has driven efforts to study the percutaneous application of ZnO NPs to diseased skin; however, in-depth studies of toxic effects on melanocytes under conditions of epidermal barrier dysfunction remain lacking.

**Methods:**

Epidermal barrier dysfunction model mice were continuously exposed to a ZnO NP-containing suspension for 14 and 49 consecutive days in vivo. Melanoma-like change and molecular mechanisms were also verified in human epidermal melanocytes treated with 5.0 µg/ml ZnO NPs for 72 h in vitro.

**Results:**

ZnO NP application for 14 and 49 consecutive days induced melanoma-like skin lesions, supported by pigmented appearance, markedly increased number of melanocytes in the epidermis and dermis, increased cells with irregular nuclei in the epidermis, recruited dendritic cells in the dermis and dysregulated expression of melanoma-associated gene *Fkbp51*, *Trim63* and *Tsp 1*. ZnO NPs increased oxidative injury, inhibited apoptosis, and increased nuclear factor kappa B (NF-κB) p65 and Bcl-2 expression in melanocytes of skin with epidermal barrier dysfunction after continuously treated for 14 and 49 days. Exposure to 5.0 µg/ml ZnO NPs for 72 h increased cell viability, decreased apoptosis, and increased Fkbp51 expression in melanocytes, consistent with histological observations in vivo. The oxidative stress–mediated mechanism underlying the induction of anti-apoptotic effects was verified using the reactive oxygen species scavenger N-acetylcysteine.

**Conclusions:**

The entry of ZnO NPs into the stratum basale of skin with epidermal barrier dysfunction resulted in melanoma-like skin lesions and an anti-apoptotic effect induced by oxidative stress, activating the NF-κB pathway in melanocytes.

**Graphical Abstract:**

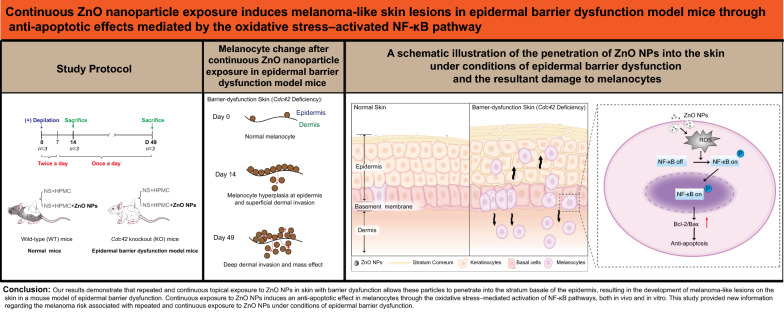

**Supplementary Information:**

The online version contains supplementary material available at 10.1186/s12951-022-01308-w.

## Introduction

Zinc oxide nanoparticles (ZnO NPs) are well known and widely used in many dermatological preparations and dermo-cosmetic products, such as sunscreens, due to their opacifying, antimicrobial, and ultraviolet (UV)-protective properties [1]. The utilization of ZnO NPs has increased due to their good dispersion quality, transparency, and non-comedogenic properties. ZnO NPs with a diameter between 20 and 100 nm are incorporated into sunscreen formulations [2]. However, NPs exhibit unique physicochemical properties and are often more toxic than their non-nanoscale counterparts [3, 4]. Therefore, great attention has been paid to the hazardous properties of ZnO NPs applied to the skin. Investigations of skin penetration have demonstrated that ZnO NPs typically do not infiltrate the epidermis [5–7] due to the strong barrier function of healthy skin, which prevents the infiltration of external materials into the epidermis; however, certain pathological skin conditions accompanied by epidermal barrier injury, such as eczema and UV damage, are at increased risk of penetration by topically applied metal oxide NPs [8, 9]. Ilves et al. suggested that atopic dermatitis (AD)-like skin enabled partial penetration of ZnO NPs through the damaged epidermis into the viable layers of the skin [1]. The physical skin barrier is mainly localized in the stratum corneum, which plays a critical role in preventing damage due to environmental stress [10]. Zhang et al. found that *Cdc42* played crucial roles in regulating the balance between keratinocyte proliferation and differentiation and in the integrity of cell–cell junctions in epidermal development. *Cdc42* is essential for epidermal barrier formation, and *Cdc42* deficiency might be associated with skin barrier damage or dysfunction [11].

Reports have suggested that ZnO NPs in sunscreens can penetrate injured or diseased skin, such as skin with sunburn, acne, or eczema, and skin with psoriasis-like features [1, 12]. Some studies have shown that NPs deposited in target organs can generate excessive oxidative stress, triggering injurious responses, such as apoptosis and DNA damage [13, 14]. Kocbek et al. showed that ZnO particles stimulated the reactive oxygen species (ROS) production inside keratinocytes [15]. Accordingly, keratinocyte viability was reduced by ZnO NPs with the concentration beyond 15 µg/ml. After 3 months, NPs were found to be present in an aggregated state within the cell cytoplasm, causing altered cell morphology. Cancer is commonly characterized by the deregulation of apoptotic cell death machinery, and changes in apoptosis can promote tumor development [16]. The upregulation of oxidative stress induced by the penetration of ZnO NPs may affect apoptotic pathways, resulting in increased carcinogenesis and malignant transformation.

Melanocytes are located in the basal layer of the epidermis and are characterized by their capacity to produce melanin, which protects the skin from damaging UV irradiation. Melanocytes are particularly sensitive to cellular damage because they are constantly exposed to multiple environmental factors, including solar radiation, which can result in the development of a continuous, low-grade oxidative state. Melanin can serve as both an antioxidant and an oxidant, depending on its redox state [17]. Oxidative stress has been shown to be involved in all stages of carcinogenesis and malignant melanocyte transformation [18–20]. However, the effects of these stress reaction reduced by ZnO NPs on melanocytes have not been studied until now. The abnormal proliferation of melanocytes in the epidermis or dermis leads to skin hyperpigmentation, accompanied by random and discontinuous cytological atypia, which is characteristic of the malignant transformation of melanocytes [21]. Melanomas are among the most immunogenic tumors, and result in the recruitment of innate immune cells, and changes of immune cells could improve the progression of malignant melanoma [22].

The balance between pro-apoptotic and anti-apoptotic protein regulators is a critical determinant for whether a cell undergoes apoptosis, and the dysregulation of apoptotic pathways is considered an underlying mechanism associated with cancer development [23, 24]. Bcl-2 serves as an “apoptotic switch,” and Bcl-2 family members can be classified as either anti-apoptotic, such as Bcl-2 and Bcl-xL, or pro-apoptotic, such as Bax and Bak, depending on their functions [25]. Melanocyte transformation is accompanied by high levels of ROS, particularly superoxide, which can activate nuclear factor kappa B (NF-κB) [26]. During melanocyte transformation, the disorganization of the melanosomal structure has been proposed to disrupt the response to ROS within the matrix, allowing the leakage of free radicals into the cytoplasm and leading to intracellular oxidative stress. Increased oxidative stress levels trigger the activation of NF-κB and other transcription factors to initiate a downstream stress response, including the induction of anti-apoptotic pathways [27].

In this study, a keratinocyte-specific *Cdc42* knockout (KO) mouse model of epidermal barrier dysfunction was used to examine the effects of continuous ZnO NP exposure on epidermal melanocytes in skin with damaged barrier integrity. The aim of this study was to investigate the influence of ZnO NPs on melanocytes under conditions associated with a high degree of epidermal barrier dysfunction, such as those caused by injury or disease, to determine whether ZnO NPs can induce malignant melanocyte transformation following penetration through damaged skin.

## Materials and methods

### Preparation and characterization of ZnO NP suspensions

ZnO NPs were purchased from Sigma-Aldrich (St. Louis, MO, USA). The morphology and size of the particles were characterized by transmission electron microscopy (TEM) (2100F, JEOL, Tokyo, Japan). The X-ray diffraction (XRD) pattern was recorded using a Rigaku Miniflex-II x-ray diffractometer (Tokyo, Japan).

To prepare a suspension for use in animal experiments, ZnO NPs were dispersed in normal saline (NS) containing 1% hydroxypropyl methylcellulose (HPMC) [12] at a concentration of 40 mg/ml. HPMC was used as a suspending agent to ensure that the NPs were uniformly dispersed and to increase operability. The suspension was ultrasonically dispersed in an ice bath for 30 min before each use.

To prepare a stock solution for the cell experiments, ZnO NPs were dispersed in phosphate-buffered saline (PBS) at a concentration of 2 mg/ml and autoclaved. The working solution was the stock solution diluted with melanocyte medium and was ultrasonically dispersed in an ice bath for 15 min before each use [12].

### Animals and treatments

C57BL/6 J mice expressing Cre recombinase under the control of the K5 proximal promoter used in the study were obtained from Dr Xiao Yang’s laboratory at Academy of Military Medical Sciences (Beijing, China). The *Cdc42*^*flox/flox*^ homozygous transgenic C57BL/6J mice used in this study were gifts from Yi Zheng at the University of Cincinnati (Cincinnati, OH, USA). Animals were housed under specific pathogen-free conditions in the animal facility at Southern Medical University Laboratory Animal Center Research Foundation. All animal studies were approved by the Bioethics Committee of Southern Medical University (No. L2018133).

**Fig. 1:**
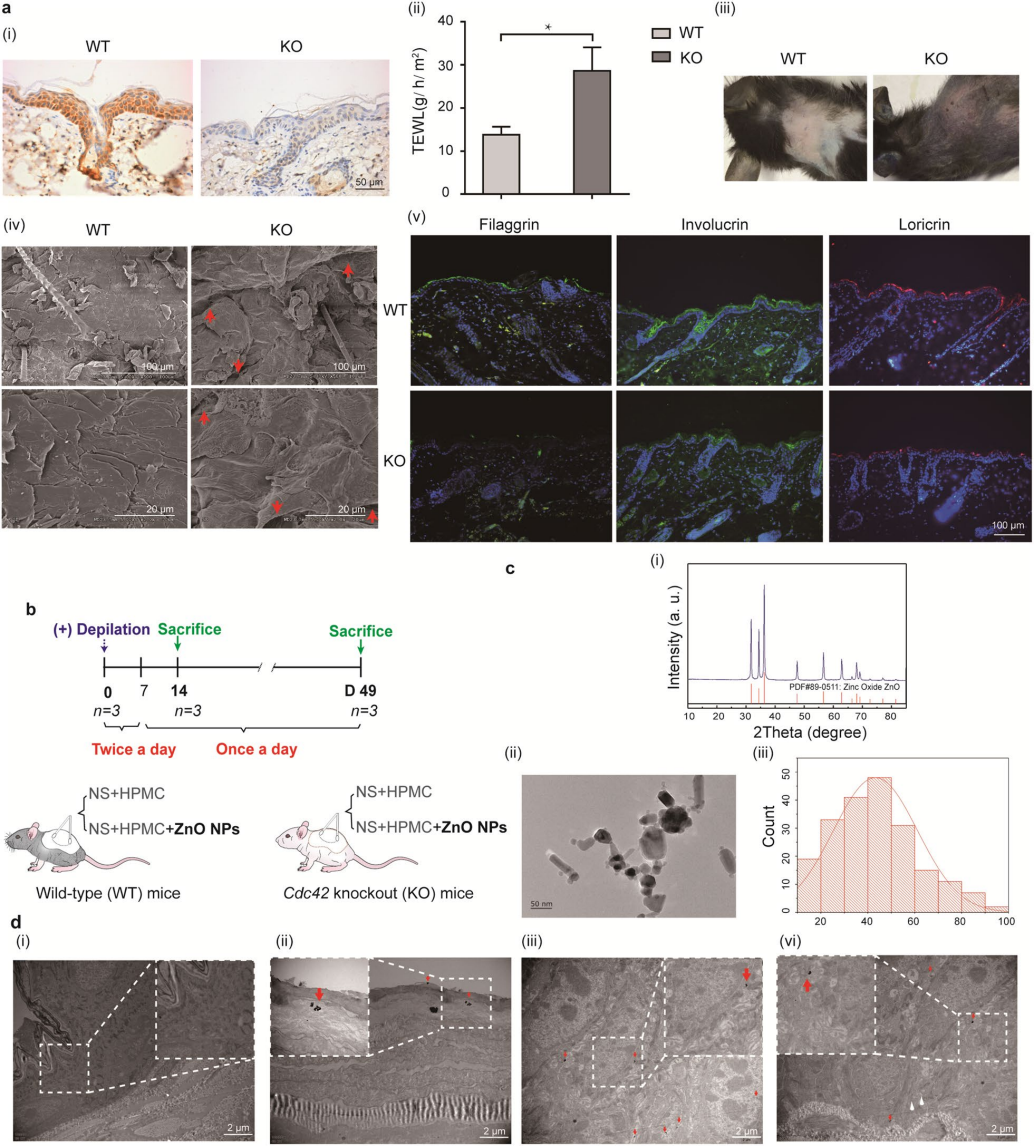
The morphology of ZnO NPs and their translocation into the skin after topical application. **a** Epidermal barrier dysfunction caused by *Cdc42* KO. i) immunohistochemistry image of *Cdc42* expression in skin of WT and *Cdc42* KO mice. Scale bar = 50 μm. ii) TEWL measurement. Data represents Mean ± SD (n = 3). *p < 0.05. iii) Skin permeability assay using X-gal staining in WT and *Cdc42* KO mice. iv) SEM of dorsal skin of WT and *Cdc42* KO mice. Scale bars = 100 or 20 μm. v) Immunofluorescence image of filaggrin, loricrin, and lnvolucrin expression in the skin of WT and *Cdc42* KO mice. Scale bar = 100 µm. **b** Experimental design for the ZnO NP treatment of WT and *Cdc42* KO mice. n = 3. **c** Characterization of ZnO NPs. i) The XRD spectrum of NPs compared against the reference material, zincite (red lines). ii) The primary size and morphology of ZnO NPs. Scale bar = 50 nm. iii) Size distribution of ZnO NPs. Particle measurements (n = 207) were performed on individual ZnO NPs. **d** i) TEM of skin tissue from WT mice after 14 days of continuous treatment with ZnO NPs. Scale bar = 2 µm. TEM of skin tissue from *Cdc42* KO mice after 14 days of continuous treatment with ZnO NPs. The presence of ZnO NPs is denoted by red arrows. ZnO NPs were detected in the stratum corneum (ii), the stratum spinosum (iii), and the stratum basale (iv). Scale bar = 2 µm. Region of Interest (ROI) at × 4 magnification

12-week-old female *Cdc42* KO mice weight 21.3 ± 0.7 g were used as a model of epidermal barrier dysfunction. After the successful establishment of the model was histologically confirmed, mice were randomly distributed into four groups containing three mice per group: wild-type (WT) and *Cdc42* KO mice were divided into negative control and ZnO NP treatment groups. The protocol used for the percutaneous application of ZnO NPs to mice is shown in Fig. 1b. A 10-cm^2^ area of the dorsal skin was shaved, and a ZnO NP suspension was applied at a dose of 2 mg/cm^2^ [28]. A 500-μL aliquot of NS (containing 1% HPMC) or a ZnO NP suspension (consisting of NS, r1% HPMC, and ZnO NPs) was topically applied twice daily for 7 days and once daily from days 8–49 for both WT and *Cdc42* KO mice [29]. Three mice were anaesthetized with 1% pentobarbital (0.1 ml per 20 g i.p.) at each time point before obtaining skin tissue. The skin tissue were cut into 1-cm^2^ full-thickness pieces and washed three times with PBS. The freshly collected skin were fixed or frozen.

### Cell culture and treatments

Human epidermal melanocytes (HEMs) were purchased from Sciencecell (Carlsbad, CA, USA) and maintained in melanocyte medium containing 1% melanocyte growth supplement (Carlsbad, CA, USA), 0.5% fetal bovine serum (Carlsbad, CA, USA), and 1% penicillin/streptomycin (Carlsbad, CA, USA) at 37 °C in a humidified atmosphere of 5% CO_2_. The culture medium was replaced every other day, and cells were passaged upon reaching approximately 90% confluence. HEMs were cultured in diluted ZnO NP working solution containing 2.5, 5.0, 7.5, or 10 µg/ml ZnO NPs for 24, 48 and 72 h.

*N*-acetylcysteine (NAC) (Sigma-Aldrich; 20 mM), the *N*-acetyl derivative of cysteine, inhibits ROS in HEMs were treated with 5.0 µg/ml ZnO NPs for 24, 48 and 72 h.

### TEM and scanning electron microscopy (SEM) observation of skin tissue

Skin tissue samples (1 × 2 mm^2^ in area) were cut from the central area of freshly excised dorsal skin using a sharp surgical blade. Samples were immersed in 2.5% glutaraldehyde and incubated overnight at 4 °C. The samples were then embedded, and ultrathin slices were prepared. The nanoparticle localization was evaluated using TEM (H-7500, Hitachi, Japan). Skin tissue was evaluated using SEM (S-3000-N, Hitachi, Ltd., Japan).

### Histopathological examination of skin tissue and HEMs

Freshly excised dorsal skin pieces were immersed in 4% paraformaldehyde for 24 h and embedded in paraffin after dehydration through graded ethanol and xylene solutions. Sections were prepared at a thickness of 4 µm. Hematoxylin and eosin staining was performed for histological analysis. Immunohistochemical analysis was used to detect the numbers of cells labeled with deoxynucleotidyl transferase dUTP nick end labeling (TUNEL) staining (Beyotime Biotechnology, China), and antibodies against CD4 (1:300, Immunoway, China), IL2RA (CD25, 1:100, ABclonal, US), CD68 (1:1000, Proteintech, US), CD80 (1:300, Proteintech, US), CD86 (1:100, ABclonal, US), CD163 (1:2000, Proteintech, US), Ki67 (1:200, Abcam, UK), 8-hydroxydeoxyguanosine (8-OHdG) (1:1000, JaICA, Japan) and Tyrosinase (Tyr) (1:250, Santa Cruz Biotechnology, Santa Cruz, CA, US) were used to detect the expression of these proteins. Images were captured using a biological microscope (DM40008, Leica, Germany). The number of positive cells per sample in epidermis was assessed by Image J software. Ten representative randomly chosen, nonadjacent, nonoverlapping fields at × 400 magnification were used for positive cell counting. The positive cell counts were recorded as cells / 0.01 mm^2^ per unit area.

### Measurements of oxidative stress biomarkers in vivo

Skin tissues that had been frozen in liquid nitrogen and stored at − 80 °C were homogenized in precooled NS using a tissue homogenizer. The homogenates (1:10 w/v) were centrifuged at 1006 × g for 10 min at 4 °C to obtain supernatants. Cells were lysed and collected. The protein concentrations of the samples were determined using a BSA kit (GBCBIO, China). The levels of catalase (CAT), glutathione (GSH), total superoxide dismutase (T-SOD) and malondialdehyde (MDA) were determined using commercial kits (Nanjing Jiancheng Bioengineering Institute, Nanjing, China), according to the manufacturer’s instructions.

### RNA sequencing (RNA-seq)

RNA from the skin of *Cdc42* KO and WT mice after ZnO NP or control treatment for 14 days were prepared for RNA-seq (three biological replicates for each group). RNA-seq experiments were performed by Novogene (Beijing, China). Briefly, total RNA was isolated from fresh skin tissue using TRIzol. mRNA was then purified from total RNA using poly-T oligo-attached magnetic beads. Sequencing libraries were generated using NEBNext® UltraTM RNA Library Prep Kit for Illumina® (NEB, USA) following manufacturer’s recommendations, and index codes were added to attribute sequences to each sample. The clustering of the index-coded samples was performed on a cBot Cluster Generation System using TruSeq PE Cluster Kit v3-cBotHS (Illumina) according to the manufacturer’s instructions. After cluster generation, the library preparations were sequenced on an Illumina HiSeq platform and 125/150 bp paired-end reads were generated. For the data analysis, raw data (raw reads) in fastq format were first processed through in-house Perl scripts. Clean data (clean reads) were obtained by removing reads containing adapters, reads containing poly-N, and low-quality reads from raw data. Reference genome and gene model annotation files were downloaded from genome website directly. An index of the reference genome was built using STAR, and paired-end clean reads were aligned to the reference genome using STAR (v2.5.1b). STAR uses the method of Maximal Mappable Prefix (MMP). HTSeq v0.6.0 was used to count the read numbers mapped to each gene. And then FPKM of each gene was calculated based on the length of the gene and reads count mapped to this gene. Differential expression analysis of three biological replicates per condition was performed using the DESeq2 R package (1.10.1). DESeq2 provides statistical routines for determining differential expression in digital gene expression data using a model based on the negative binomial distribution. The resulting P-values were adjusted using the Benjamini and Hochberg approach for controlling the false discovery rate. Genes with an adjusted P-value < 0.05 found by DESeq2 were assigned as differentially expressed. Analysis of differential expression was performed using the edgeR R package (3.12.1). The P values were adjusted using the Benjamini and Hochberg method. The hierarchical clustering heat map was generated with the ggplot library. The sequencing data are available through the GEO database with accession number GSE183662.

### Quantitative real-time reverse transcriptase PCR (qRT-PCR) analysis in vivo and in vitro

Skin tissue that had been frozen in liquid nitrogen and stored at − 80 °C was homogenized in liquid nitrogen and extracted with TRIzol reagent (Thermo Fisher Scientific, Waltham, MA, USA), according to the manufacturer’s instructions. The concentration and purity of total RNA were determined by measuring the absorbance at 260 and 280 nm using a spectrophotometer (Molecular Devices, San Jose, CA, USA). Complementary DNA (cDNA) was reverse transcribed from mRNA samples using a PrimeScript™ RT Reagent Kit (TaKaRa Bio, Shiga, Japan). qRT-PCR was performed with a LightCycler 480 Sequence Detector System (Roche, Switzerland) using a commercial kit (SYBR Premix Ex Taq II, TaKaRa Bio). The cells were lysed with TRIzol reagent, and mRNA extraction, cDNA synthesis, and qRT-PCR were performed as described for skin tissue. The mouse and human primer sequences used for qRT-PCR are shown in Tables 1 and 2, respectively.

**Table 1:** Mouse-specific primer sequences used in the qRT-PCR analysis

Gene	Forward primer	Reverse primer
*Fkbp51*	CAAACCCAAACGAAGGAGCAACG	TTCCCCAACAACGAACACCACATC
*Trim63*	GTCATCCTGCCCTGCCAACA	GCAACGGAAACGACCTCCAGAC
*Tsp1*	GGCCGAGGTGTCGAACATG	GGTCACTGTAGTGACCCAGGTAGT
*Gapdh*	AGAAGGTGGTGAAGCAGGCATC	CGAAGGTGGAAGAGTGGGAGTTG

**Table 2:** Human-specific primer sequences used in the qRT-PCR analysis

Gene	Forward primer	Reverse primer
FKBP51	ATGGGAAGATAGTGTCCTGGTTAGA	CCTTGGCTGACTCAAACTCGT
GAPDH	AGGTCGGTGTGAACGGATTTG	TGTAGACCATGTAGTTGAGGTCA

### Cell survival assay

The cells were seeded in 96-well plates at a density of 3 × 10^4^ cells per well and allowed to attach overnight. The cells were treated with 2.5, 5.0 and 7.5 µg/ml ZnO NPs for 24, 48 and 72 h. Cell viability was determined using a cell-counting 8 (CCK-8) assay kit according to the manufacturer’s instructions (Dojindo Molecular Technologies, Kumamoto, Japan), and the absorbance was measured at a wavelength of 450 nm using a microplate reader.

### Annexin V/PI analysis

The cells were seeded in 24-well plates at a density of 1.5 × 10^5^ cells per well and allowed to attach overnight. The cells were treated with 5.0 µg/ml ZnO NPs or culture medium for 24, 48 and 72 h. Cells were washed, digested with EDTA-free trypsin and stained with FITC-conjugated Annexin V and PI (Beyotime, China) according to the manufacturer’s instructions. The final cell suspensions in PBS were analyzed using flow cytometry (FACSAria III, BD, US).

### Cellular ROS assay

The cells were seeded in 24-well plates at a density of 1.5 × 10^5^ cells per well and allowed to attach overnight. The cells were treated with 5.0 µg/ml ZnO NPs or culture medium for 24, 48 and 72 h. Cellular ROS production was determined by using 2′,7′ -dichlorofluorescein diacetate (DCFH-DA) probes (Nanjing Jiancheng Bioengineering Institute). The treated cells were washed, collected with EDTA-free trypsin, and incubated with the probes (10 µM) for 30 min at 37 °C, washed twice with PBS, and resuspended in 500 µL PBS. The fluorescence intensity of the cells was analyzed using a flow cytometer (FACSAria III, BD, San Jose, CA, USA) at excitation/emission wavelengths of 485/535 for DCFH-DA.

### Immunofluorescence examination in vivo and vitro

Sections treated for immunofluorescence staining, Antibodies against Tyr (1:300, Santa Cruz Biotechnology, Santa Cruz, CA, US), Tyrosinase-related protein-2/dopachrome tautomerase (Trp-2/Dct) (1:200, LifeSpan BioSciences, Seattle, WA, USA), Fkbp51 (1:200, Santa Cruz Biotechnology), NF-κB p65 (1:500, Cell Signaling Technology, US) and Bcl-2 (1:200, Cell Signaling Technology, US) were used to detect the expression of these proteins in vivo study. Images were captured using a biological microscope (DM40008, Leica, Germany). The proportion of positive staining cells in epidermis per sample was assessed by Image J software. Ten representative randomly chosen, nonadjacent, nonoverlapping fields at × 400 magnification per area of epidermis were used for positive cell counting. The positive cell counts were recorded as % Area.

The 1.5 × 10^5^ cells were seeded on coverslips, treated with ZnO NPs as previously describe, fixed with 4% paraformaldehyde overnight, and permeabilized with 0.5% Triton X-100 for 30 min. After blocking in bovine serum albumin with 10% goat serum for 1 h, the cells were incubated with Fkbp51 and Bcl-2 (1:200 and 1:200, Santa Cruz Biotechnology, US), NF-κB p65 (1:500, Cell Signaling Technology, US) at 4 °C overnight and incubated with Alexa 488/568-conjugated secondary antibodies (Proteintech, Rosemont, IL, USA) at room temperature for 1 h. Fluorescence images were captured using a fluorescence microscope (Leica, Japan). Ten representative randomly chosen, nonadjacent, nonoverlapping fields at × 400 magnification per core were used for cell counting. The proportion of positive staining cells per sample was assessed by Image J software.

### Western blot analysis

Cells were collected and lysed using reagents from a nuclear protein and cytoplasmic protein extraction kit (Beyotime Biotechnology, China) containing 1 mM protease and phosphatase inhibitor (Beyotime Biotechnology). The cell lysates were heated in sodium dodecyl sulfate–polyacrylamide gel electrophoresis (SDS-PAGE) sample buffer (Genstar, China) at 99 °C for 5 min. The proteins were separated by SDS-PAGE and transferred to polyvinylidene difluoride membranes (Merck Millipore, Darmstadt, Germany) that were then blocked with 5% skim milk for 1 h. The membranes were incubated overnight at 4 °C with primary antibodies, including anti-NF-κB p65, anti-phospho-NF-κB (p-NF-κB) p65, and anti-Bax (1:1000, 1:1000 and 1:1000, all Cell Signaling Technology, US), anti-Bcl-2(1:500, Santa Cruz Biotechnology, US) and anti-Gapdh (1: 50,000, Proteintech, US). The membranes were then washed with Tris-buffered saline containing Tween 20 (TBST) and incubated at room temperature with horseradish peroxidase (HRP)-conjugated secondary antibodies (Cell Signaling Technology) for 1 h. Proteins were detected using an enhanced chemiluminescence (ECL) kit (WBLKS0500, Merck Millipore) and an automatic chemiluminescence image analysis system (Tanon, China). Quantitative analysis was performed using ImageJ software.

### Statistical analysis

All the data are presented as the mean ± standard deviation (SD) and were analyzed with SPSS 22.0 software. Comparisons among each group were assessed using one-way ANOVA with the Bonferroni post hoc test when the variance in the data was homogenous. Using a non-parametric test when the variance in the data was not homogenous. Differences at P < 0.05 were considered to be statistically significant.

## Results

### ZnO NPs penetrate into deeper skin layers in a mouse model of epidermal barrier dysfunction

A murine model of epidermal barrier dysfunction was used to study particle penetration in this study. In this model, epidermal barrier dysfunction is caused by *Cdc42* KO (Fig. 1a), which was confirmed by PCR (Additional file 1: Figure S1a). *Cdc42* protein was entirely absent from *Cdc42* KO mice compared WT controls (Fig. 1a-i). Transepidermal water loss (TEWL), which reflects inside-out barrier function [30], was considerably increased in *Cdc42* KO mice compared with WT mice (Fig. 1a-ii). In the X-gal staining assay [30], which reflects outside-in barrier function, the dye penetrated the skin of the *Cdc42* KO mice but not the skin of WT mice (Fig. 1a-iii). SEM revealed larger gaps between epidermal corneocytes in *Cdc42* KO mice than in WT mice (Fig. 1a-iv). Filaggrin, loricrin, and lnvolucrin are pathogenic implications in AD [31]. It was showed that decreased expression of filaggrin, loricrin, and lnvolucrin in the epidrmis of *Cdc42* KO mice than in WT mice (Fig. 1a-v). Together, our results suggested that *Cdc42* deletion resulted in both outside-in and inside-out barrier impairments.

The protocol used for the percutaneous application of ZnO NPs to mice is shown in Fig. 1b. In our study, a short 7-day period of ZnO NP application is equivalent to a week at the beach or on holiday. We examined the effects of continuous epidermal exposure to ZnO NPs for up to 14 and even 49 days, which is representative of the large numbers of people who use sunscreens on a daily basis [29].

XRD was used to determine the composition of the ZnO NPs used in this study (Fig. 1c-i), and the morphology and size distributions of ZnO NPs are shown in Fig. 1c-ii. The evaluation of individual particle diameters yielded a primary particle size distribution of 43.25 ± 17.96 nm (Fig. 1c-iii).

Although NPs are hypothesized to be unable to pass through healthy, intact skin, their ability to penetrate skin with epidermal barrier dysfunction skin remains unknown. Skin samples were collected to investigate whether ZnO NPs could be detected in the different skin layers of WT and epidermal barrier dysfunction model mice. As shown in Fig. 1d-i, a minimal number of ZnO NPs were detected in the epidermis of WT mice continuously treated with ZnO NPs after 14 days of treatment. By contrast, ZnO NPs were detected on the skin surface, and particle penetration was observed throughout the epidermis, including the stratum corneum, stratum spinosum, and stratum basale, in *Cdc42* KO mice continuously treated with ZnO NPs for 14 days (Fig. 1d-ii, iii, iv). This analysis suggested that the epidermal barrier dysfunction induced by *Cdc42* KO allowed for the penetration of ZnO NPs through the damaged epidermis into the viable layers of the epidermis.

### ZnO NPs induce the dysregulated expression of genes associated with melanoma in epidermal barrier dysfunction model mice

**Fig. 2:**
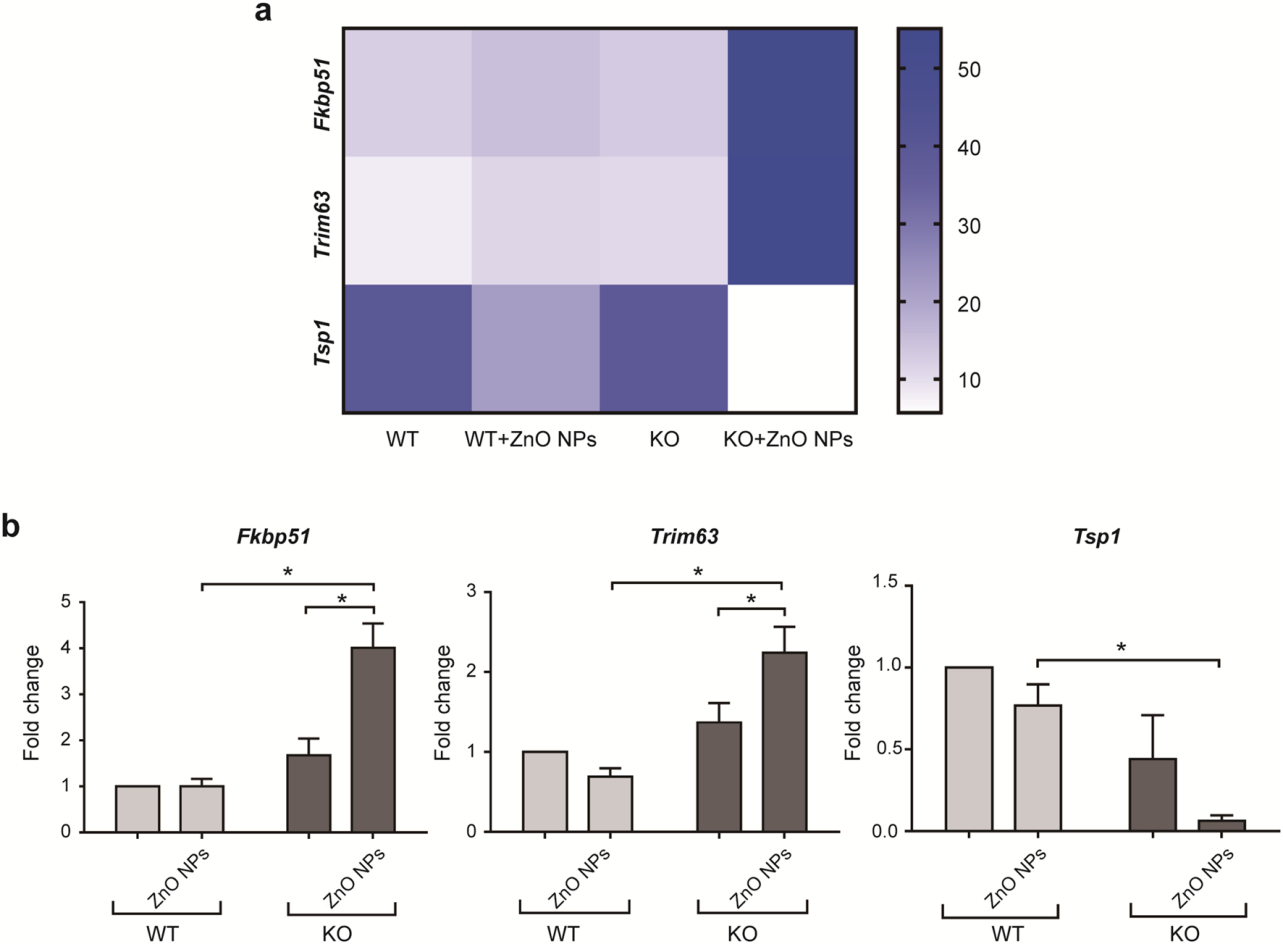
Gene expression associated with melanoma in the skin of epidermal barrier dysfunction model mice. **a** Heatmap (*p* < 0.05 or *p*_adj_ < 0.05) showing DE genes associated with melanoma after 14 days of ZnO NP treatment. **b** mRNA levels of DE genes were measured by qRT-PCR and normalized to *Gapdh* expression levels. Data represents Mean ± SD. *p < 0.05

The evaluation of changes in genomic expression represents a powerful approach for determining the response of an organism to NP exposure. RNA-seq can be used to quantify the expression of most genes in an organism by measuring the levels of RNA transcripts following NP exposure relative to the expression levels observed under normal physiological conditions (Additional file 1: Figure S2) [32]. Figure 2a shows a hierarchical clustering heatmap for three differentially expressed (DE) genes identified on day 14 when comparing expression levels between the skin of WT and *Cdc42* KO mice in the negative control group (no ZnO NP exposure) and between the skin of WT and *Cdc42* KO mice continuously treated with ZnO NPs. The transcriptome produced in response to continuous ZnO NP treatment for 14 days in the skin of *Cdc42* KO mice showed elevated levels of *FK506-binding protein 51 (Fkbp51)* and *tripartite motif-containing 63 (Trim63)* transcripts and reduced levels of *Thrombospondin-1 (Tsp1)* transcripts than in the skin of *Cdc42* KO mice in the negative control group and WT mice continuously treated with ZnO NPs.

The levels of DE genes were analyzed by qRT-PCR analysis of skin samples obtained on day 14 from WT and *Cdc42* KO mice in the negative control group and from WT and *Cdc42* KO mice continuously treated with ZnO NPs (Fig. 2b). After 14 days of treatment, higher levels of *Fkbp51* and *Trim63* mRNA were observed in the skin of *Cdc42* KO mice continuously treated with ZnO NPs than in the skin of *Cdc42* KO mice in the negative control group and the skin of WT mice continuously treated with ZnO NPs. *Tsp1* mRNA levels were lower in the skin of *Cdc42* KO mice continuously treated with ZnO NPs for 14 days than in the skin of WT mice continuously treated with ZnO NPs.

### Epidermal barrier dysfunction model mice treated continuously with ZnO NPs develop melanoma-like lesions

Patients with melanoma present with a characteristic increase in skin pigmentation, accompanied by melanocyte proliferation and infiltration into the dermis, and changes in the skin local immune microenvironment [22, 33].

**Fig. 3:**
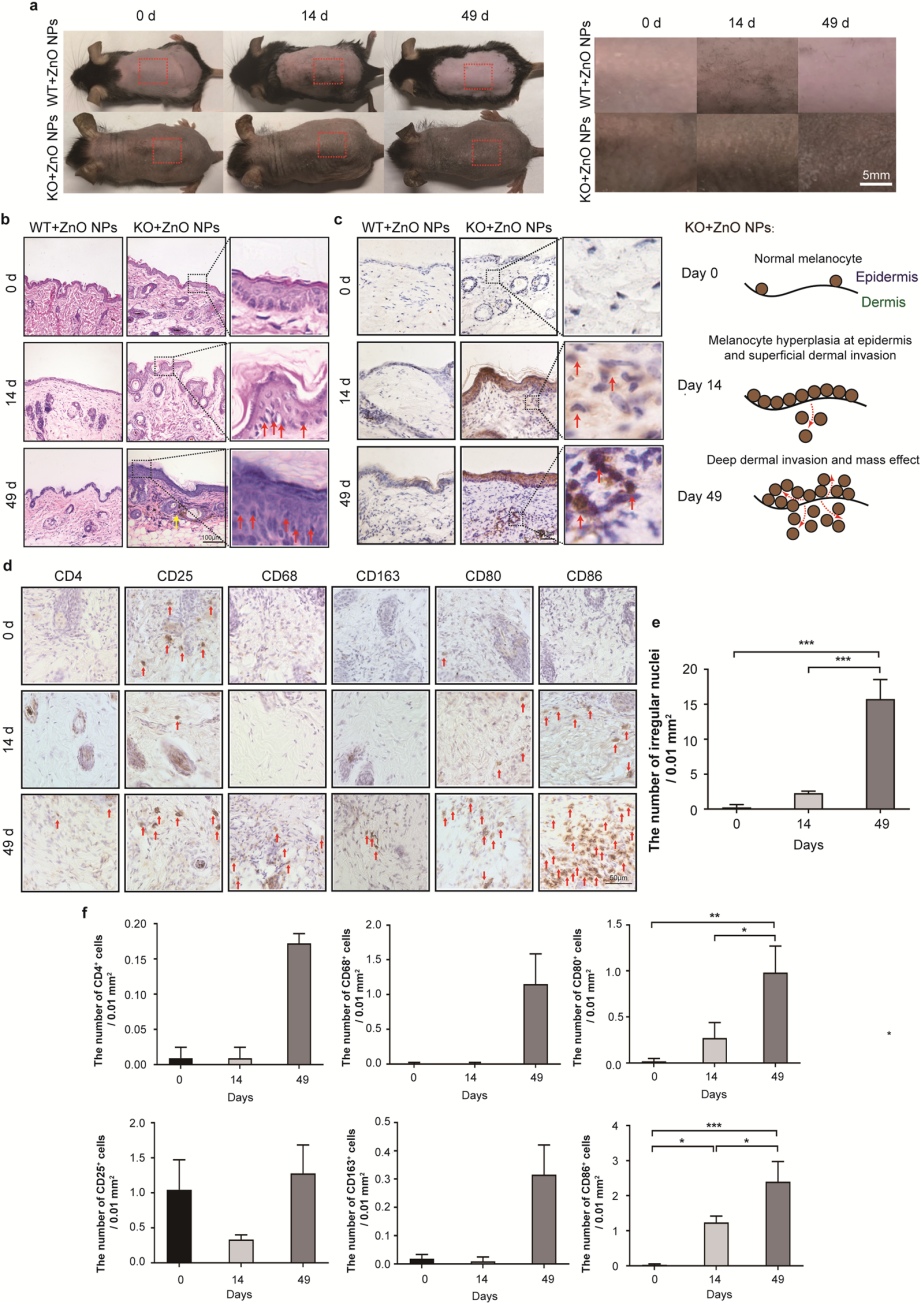

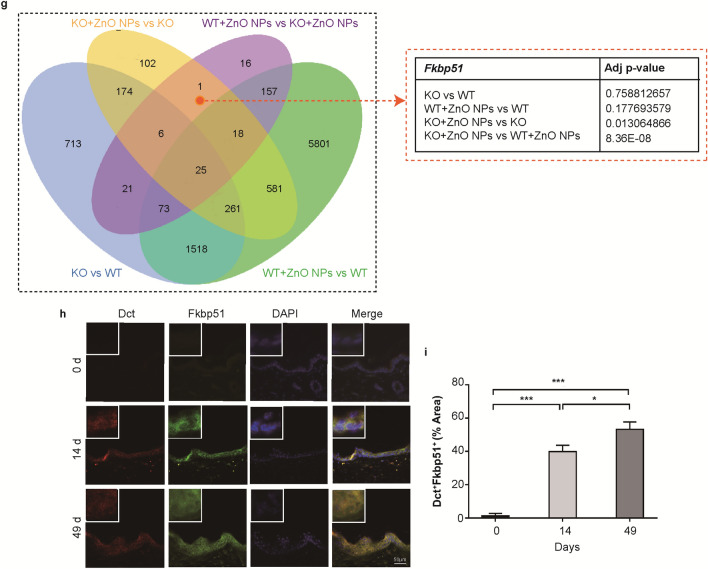
Melanoma-like skin changes observed in epidermal barrier dysfunction model mice.**a** Gross morphology of skin from *Cdc42* KO and WT mice continuously treated with ZnO NPs, observed on days 0, 14 and 49. Scale bar = 5 mm. **b** Pathologic changes in the skin tissue from WT and *Cdc42* KO mice continuously treated with ZnO NPs, observed on day 0, 14 and 49. The presence of melanin granules is denoted by yellow arrows. Scale bar = 100 µm. The presence of irregular nuclei is denoted by red arrows. **c** Immunohistochemical analysis of Tyr in skin tissue from *Cdc42* KO and WT mice continuously treated with ZnO NPs for 0, 14 and 49 days. Scale bar = 50 µm. **d** Immunohistochemistry image of CD4, CD25, CD68, CD163, CD80 and CD86 staining in the skin of *Cdc42* KO mice continuously treated with ZnO NPs for 0, 14 and 49 days. Scale bar = 50 µm. **e** Quantitative analysis of the number of irregular nuclei in the epidermis of *Cdc42* KO mice continuously treated with ZnO NPs for 0, 14 and 49 days. **f** Quantitative analysis of the number of CD4^+^, CD25^+^, CD68^+^, CD163^+^, CD80^+^ and CD86^+^ cells detected in dermis of *Cdc42* KO mice continuously treated with ZnO NPs for 0, 14 and 49 days. **g** Venn diagram (left panel; p_adj_ < 0.05) comparing the identified normalized DE genes among various between-group comparisons, revealing *Fkbp51* as associated with melanoma-like changes after 14 days of continuous ZnO NP treatment. **h** Immunofluorescence image of Dct^+^Fkbp51^+^ staining in the epidermis of *Cdc42* KO mice continuously treated with ZnO NPs for 0, 14 and 49 days. Scale bar = 50 µm. **i** Quantitative analysis of the proportion of Dct^+^Fkbp51^+^ cells detected in the epidermis of *Cdc42* KO mice continuously treated with ZnO NPs for 0, 14 and 49 days. Data represents Mean ± SD (n = 3). *p < 0.05, **p < 0.01 and ***p < 0.001

After 14 and 49 days of continuous ZnO NP treatment, an increase in skin pigmentation was observed in *Cdc42* KO mice, but no obvious change was observed in WT mice (Fig. 3a). No significant changes in gross morphology were observed in the skin of WT or *Cdc42* KO mice in the negative control group on day 14 or 49 (Additional file 1: Figure S3a).

We conducted histopathological evaluations of mouse skin samples (Fig. 3b). On days 14 and 49, the skin of WT mice treated continuously with ZnO NPs showed no obvious change. However, on days 14 and 49, the skin of *Cdc42* KO mice treated continuously with ZnO NPs was characterized by a hyperplastic epidermis, accompanied by large or irregular nuclei. The quantitative analysis (Fig. 3e) revealed that continuous ZnO NP treatment for 49 days increased the number of irregular nuclei in the epidermis of *Cdc42* KO mice compared with 0 and 14 days. Obvious melanin granules were found in a deeper layer of the dermis in skin samples from *Cdc42* KO mice treated continuously with ZnO NPs for 49 days. No significant histological changes were observed in the skin of either WT or *Cdc42* KO mice in the negative control group when examined on either day 14 or 49 (Additional file 1: Figure S3b).

Tyr is an enzyme that hydroxylates tyrosine, which represents the first step in the synthesis of melanin. In melanoma, Tyr can be observed as fine, granular, cytoplasmic staining, and positive staining tends to be strong and diffuse [34]. Negligible amounts of positive Tyr staining were observed in the skin of WT and *Cdc42* KO mice in the negative control group on days 14 and 49 (Additional file 1: Figure S3c). As shown in Fig. 3c, no significant Tyr-positive cells were observed in the epidermis or dermis of WT mice continuously treated with ZnO NPs for 14 or 49 days. However, distinctly positive Tyr cells were detected in the epidermis of *Cdc42* KO mice continuously treated with ZnO NPs for 14 and 49 days, and slightly positive Tyr cells were found in the dermis of *Cdc42* KO mice continuously treated with ZnO NPs for 49 days.

Helper CD4^+^ T cells involve the activation and expansion of adaptive immune cells in melanomas. Few CD4 + staining cells were observed in the dermis of *Cdc42* KO mice after continuous ZnO NP treatment for 0 and 14 days. Slightly CD4^+^ staining cells were observed in the dermis of *Cdc42* KO mice after continuous ZnO NP treatment for 49 days (Fig. 3d). Melanoma growth and progression have been correlated with the presence of CD4^+^CD25^+^ regulatory T cells (Treg) [35]. Slightly CD25^+^ staining cells were observed in the dermis of *Cdc42* KO mice after continuous ZnO NP treatment for 0, 14 and 49 days (Fig. 3d).

Melanoma cells recruit and modify the function of macrophages (M) within the TME. Tumor-associated macrophages (TAM) are frequently polarized toward a M2 phenotype, which express CD68 and CD163 [36]. Few CD68^+^ and CD163^+^ staining cells were observed in the dermis of *Cdc42* KO mice after continuous ZnO NP treatment for 0 and 14 days. Slightly CD68^+^ and CD163^+^ staining cells were observed in the dermis of *Cdc42* KO mice after continuous ZnO NP treatment for 49 days (Fig. 3d).

Dendritic cells (DC) are among the most efficient in eliciting cytotoxic T cell responses against infection and malignancy. Mature DCs express a plethora of co-stimulatory markers, including CD80 and CD86 (cluster of differentiation 80 and 86, respectively), which are essential for activation of melanoma-specific T cells [37, 38]. Few positive staining cells were observed in the dermis of *Cdc42* KO mice on day 0. However, increased numbers of CD80^+^ and CD86^+^ staining cells were observed in dermis of *Cdc42* KO mice after 14 or 49 days of continuous ZnO NP treatment (Fig. 3d). The quantitative analysis revealed that continuous ZnO NP treatment for 14 days increased the number of CD86^+^ staining cells in the dermis of *Cdc42* KO mice than 0 day, and the number of CD80^+^ and CD86^+^ staining cells increased further after treatment for 49 days (Fig. 3f).

Figure 3g shows a Venn diagram of the DE genes identified in various group comparisons, revealing *Fkbp51* as a DE gene when comparing gene expression in the skin on day 14 between the *Cdc42* KO mice in the negative control group and the *Cdc42* KO mice continuously treated with ZnO NPs and between the WT and *Cdc42* KO mice continuously treated with ZnO NPs.

Dct is a melanocyte marker [39]. To investigate whether melanocytes are the primary source of *Fkbp51* expression in the skin, skin tissue from *Cdc42* KO mice continuously treated with ZnO NPs was assessed for the presence of Dct^+^Fkbp51^+^ cells by immunofluorescence labeling (Fig. 3h). Few positive staining cells were observed in the skin of *Cdc42* KO mice on day 0. However, in samples from *Cdc42* KO mice after 14 or 49 days of continuous ZnO NP treatment, increased staining of both Dct^+^ and Fkbp51^+^ cells were observed, and overlaying these images revealed that most of these cells were Dct^+^Fkbp51^+^. The quantitative analysis (Fig. 3i) revealed that continuous ZnO NP treatment for 14 days increased Dct^+^Fkbp51^+^ staining in the epidermis of *Cdc42* KO mice compared with day 0, and the level of Dct^+^Fkbp51^+^ staining increased further after 49 days of treatment.

### ZnO NP treatment significantly increases oxidative injury in the skin of a mouse model of epidermal barrier dysfunction

**Fig. 4:**
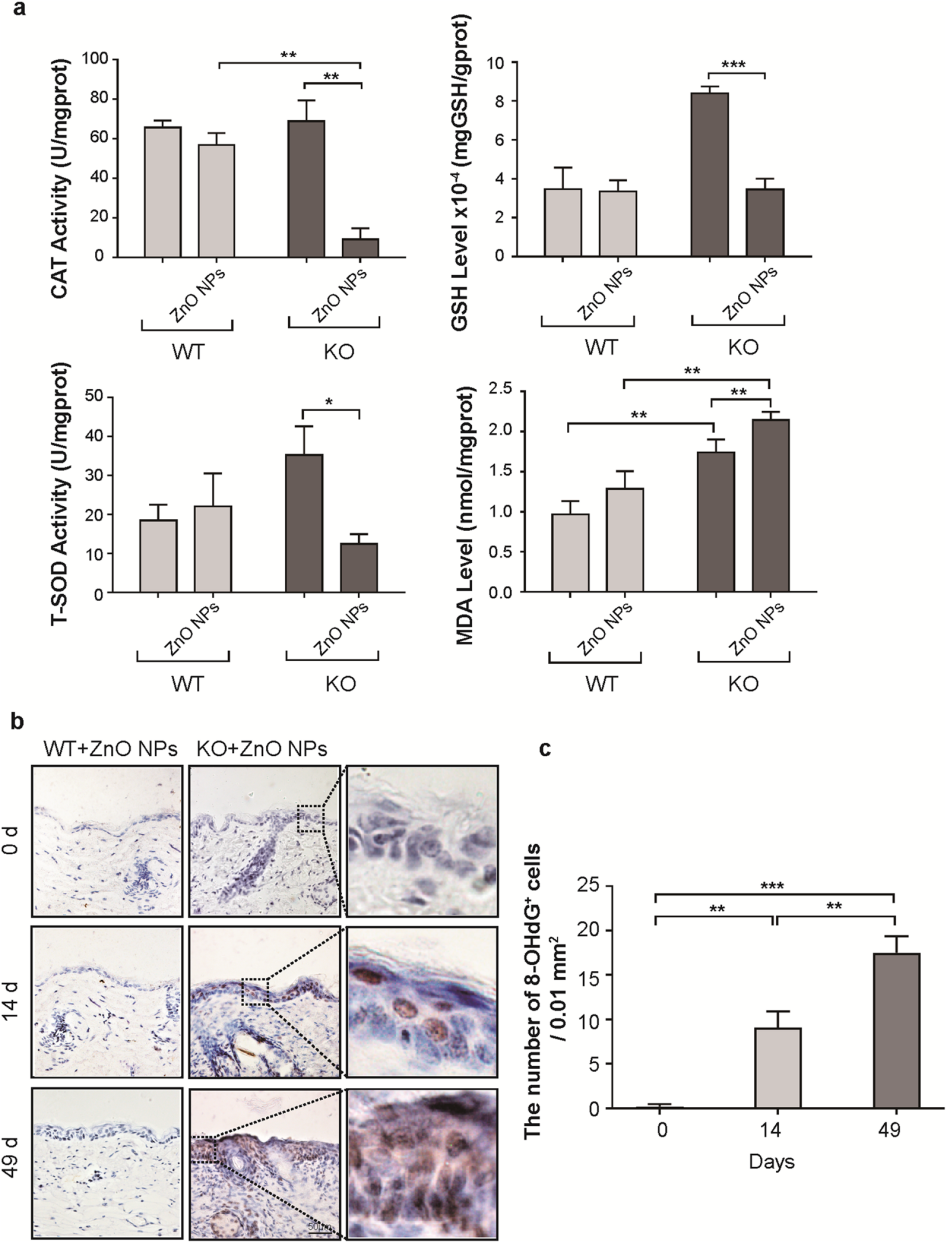
Oxidative stress injury in skin tissue **a** Changes in the levels of oxidative stress markers CAT, GSH, T-SOD, and MDA in skin tissue. **b** Immunohistochemistry image of 8-OHdG staining in the skin of *Cdc42* KO mice continuously treated with ZnO NPs for 0, 14 and 49 days. Scale bar = 50 µm. **c** Quantitative analysis of the number of 8-OHdG^+^ cells in the epidermis of *Cdc42* KO mice continuously treated with ZnO NPs for 0, 14 and 49 days. Data represents Mean ± SD (n = 3). *p < 0.05, **p < 0.01 and ***p < 0.001

Oxidative stress activates kinases to maintain the balance of intracellular redox reactions. The expression or activity levels of CAT, GSH, T-SOD, MDA, and 8-OHdG in skin tissue were determined to assess the oxidative response to treatment with ZnO NPs (Fig. 4a). Compared with skin from *Cdc42* KO mice in the negative control group and WT mice continuously treated with ZnO NPs, the CAT level decreased in the skin of *Cdc42* KO mice continuously treated with ZnO NPs after 14 days. The skin of *Cdc42* KO mice continuously treated with ZnO NPs also displayed reduced GSH and T-SOD activity after 14 days than *Cdc42* KO mice in the negative control group. MDA levels increased in the skin of *Cdc42* KO mice compared with WT mice in the negative control group, and the MDA levels were higher in the skin of *Cdc42* KO mice continuously treated with ZnO NPs than in the skin of *Cdc42* KO mice in the negative control group when assessed on day 14.

As shown in Fig. 4b, minimal positive 8-OHdG staining was detected in the epidermis of *Cdc42* KO mice at day 0, whereas a moderate number of 8-OHdG–positive cells could be observed following 14 days of continuous ZnO NP treatment, and a marked number of 8-OHdG–positive cells were observed after 49 days of treatment. The quantitative analysis (Fig. 4c) revealed an increase in 8-OHdG^+^ nuclei in the epidermis of *Cdc42* KO after 14 days of continuous ZnO NP treatment compared with day 0, with a further increase observed after 49 days of treatment.

### ZnO NP treatment has an anti-apoptotic effect on melanocytes in a mouse model of epidermal barrier dysfunction

**Fig. 5:**
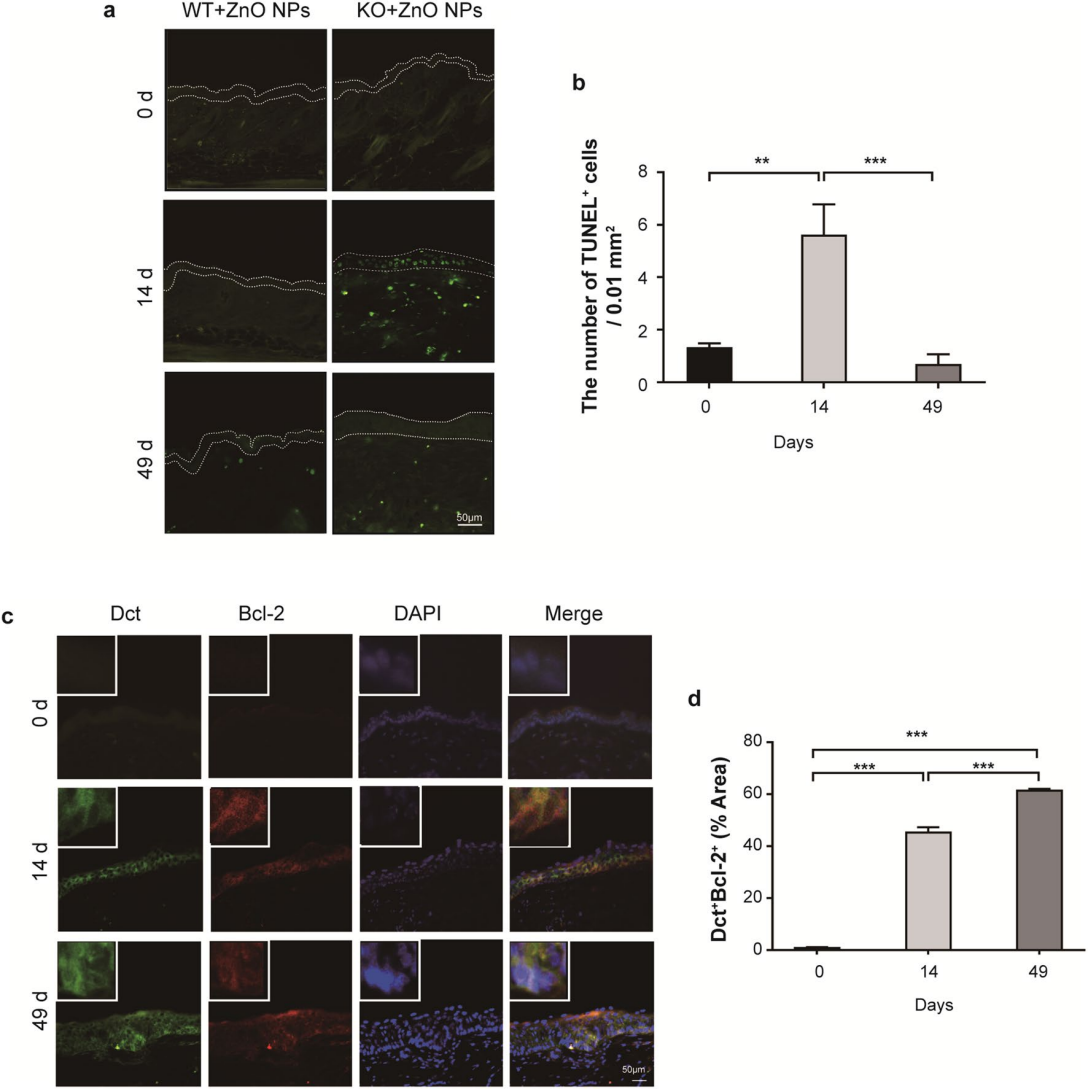
Apoptosis in skin. **a** TUNEL staining in the skin of *Cdc42* KO mice. Scale bar = 50 µm. **b** Quantitation of the number of TUNEL^+^ cells in the epidermis of *Cdc42* KO mice continuously treated with ZnO NPs for 0, 14 and 49 days. Immunofluorescence image of Dct^+^Bcl-2^+^ staining in the skin of *Cdc42* KO mice continuously treated with ZnO NPs for 0, 14, and 49 days. Scale bar = 50 µm. **d** Quantitative analysis of the proportion of Dct^+^Bcl-2^+^ detected in epidermis of *Cdc42* KO mice continuously treated with ZnO NPs for 0, 14 and 49 days. Data represents Mean ± SD (n = 3). **p < 0.01 and ***p < 0.001

The induction of apoptosis following ZnO NP treatment was assessed using the TUNEL assay (Fig. 5a). Negligible TUNEL^+^ staining was observed in the epidermis of WT and *Cdc42* KO mice on day 0. By contrast, many TUNEL^+^ cells could be observed in the epidermis of *Cdc42* KO mice after 4 days of continuous treatment with ZnO NPs (Additional file 1: Figure S4a). A moderate number of TUNEL^+^ cells could be observed in the epidermis of *Cdc42* KO mice after 14 days of continuous treatment with ZnO NPs. The skin of *Cdc42* KO mice continuously treated with ZnO NPs for 49 days showed negligible TUNEL^+^ cells. Quantitative analysis of TUNEL staining was decreased on day 14 compared with day 4 of continuous ZnO NP treatment in *Cdc42* KO (Additional file 1: Figure S4b), although quantitative analysis (Fig. 5b) revealed an increase in TUNEL staining after 14 days of continuous ZnO NP treatment in *Cdc42* KO mice compared with day 0. The level of TUNEL staining in *Cdc42* KO mice treated with ZnO NPs for 49 days was lower than the levels observed after 4 (Additional file 1: Figure S4b) and 14 days (Fig. 5b) of treatment.

The occurrence of epidermal hyperplasia was further confirmed by evaluating the expression of the proliferation marker Ki67. As shown in Figure S4c, negligible Ki67^+^ staining was observed in the epidermis of *Cdc42* KO mice on day 0. A slight increase in Ki67^+^ cells was observed in the epidermis of *Cdc42* KO mice continuously treated with ZnO NPs for 14 days, and marked Ki67^+^ staining was observed on day 49 of treatment. The quantitative analysis (Additional file 1: Figure S4d) revealed that the number of Ki67^+^ nuclei in *Cdc42* KO mice treated with ZnO NPs for 49 days increased compared with 0 and 14 days.

A negligible change in the levels of apoptosis and proliferation markers was observed in the epidermis of WT mice continuously treated with ZnO NPs. We used Bcl-2 as an anti-apoptotic marker to investigate apoptosis change in the melanocytes, and skin tissues were subjected to double immunofluorescence staining to detect Dct and Bcl-2 in the skin of *Cdc42* KO mice continuously treated with ZnO NPs (Fig. 5c). Few positive staining cells were observed in the skin of *Cdc42* KO mice on day 0. After 14 and 49 days of ZnO NP treatment, image overlay revealed that most Dct^+^ cells observed in skin from *Cdc42* KO mice were also Bcl-2^+^. The quantification of Dct^+^Bcl-2^+^ double immunofluorescence staining in *Cdc42* KO mice continuously treated with ZnO NPs (Fig. 5d) revealed a higher level of Dct^+^Bcl-2^+^ staining on day 14 compared with day 0, with a further increase observed on day 49.

### ZnO NP treatment induces NF-κB activation, accompanied by Bcl-2 expression, in melanocytes in a mouse model of epidermal barrier dysfunction

**Fig. 6:**
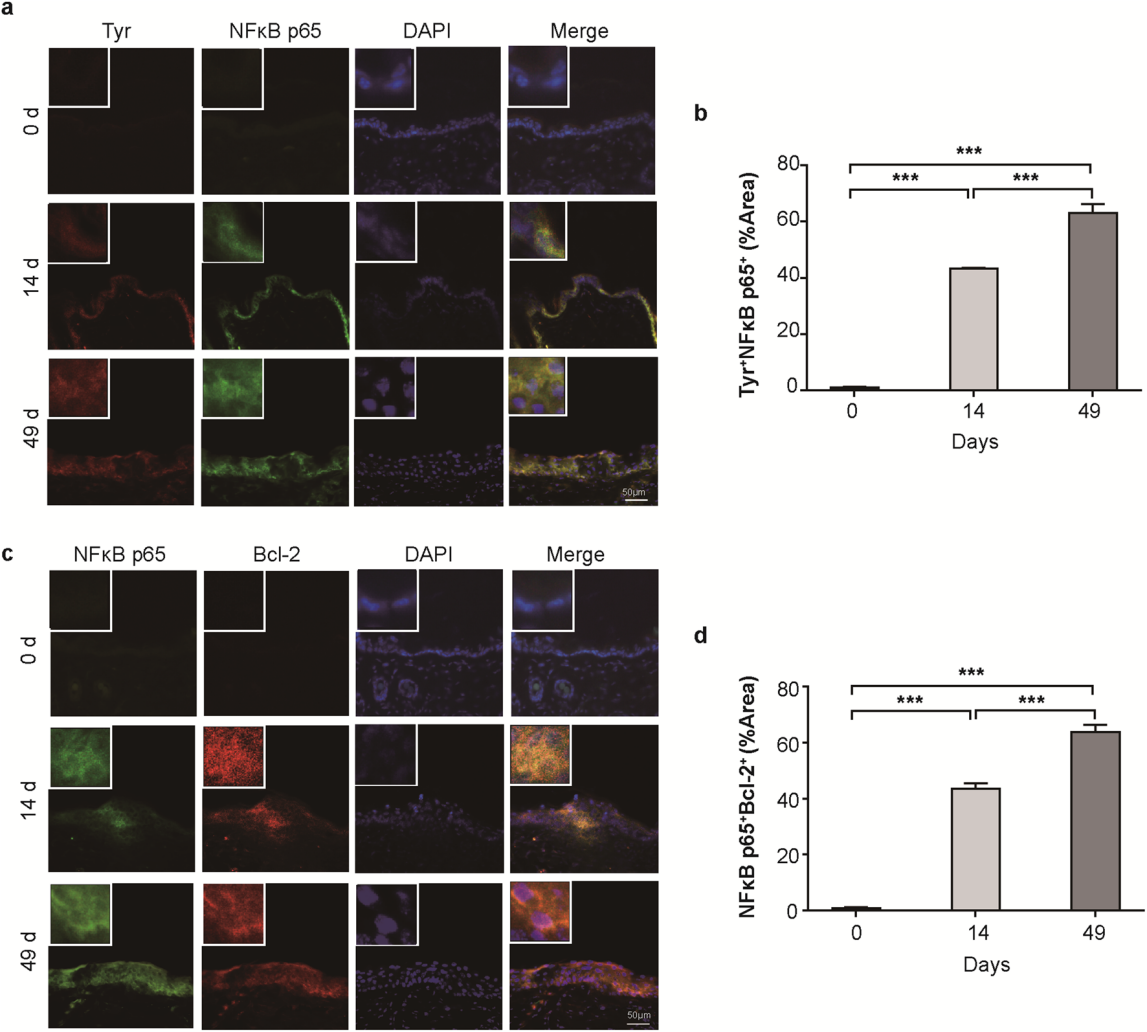
Tyr, NF-κB and Bcl-2 expression in skin. **a** Immunofluorescence image of Tyr^+^NF-κB p65^+^ staining in the skin of *Cdc42* KO mice continuously treated with ZnO NPs for 0, 14, and 49 days. Scale bar = 50 µm. **b** Quantitative analysis of the proportion of Tyr^+^NF-κB p65^+^ cells detected in the epidermis of *Cdc42* KO mice continuously treated with ZnO NPs for 0, 14 and 49 days. **c** Immunofluorescence image of NF-κB p65^+^Bcl-2^+^ staining in the skin of *Cdc42* KO mice continuously treated with ZnO NPs for 0, 14 and 49 days. Scale bar = 50 µm. **d** Quantitative analysis of the proportion of NF-κB p65^+^Bcl-2^+^ cells detected in the epidermis of *Cdc42* KO mice after continuously treated with ZnO NPs for 0, 14 and 49 days. Data represents Mean ± SD (n = 3). ***p < 0.001

To investigate whether NF-κB was activated in melanocytes, immunofluorescence analysis of Tyr^+^NF-κB p65^+^ cells was performed in skin from *Cdc42* KO mice continuously treated with ZnO NPs (Fig. 6a). Few positive staining cells were observed in the skin of *Cdc42* KO mice on day 0. Image overlay revealed that the Tyr^+^ cells observed after 14 and 49 days of continuous ZnO NP treatment in *Cdc42* KO mice were also NF-κB p65^+^. Quantitative analysis of Tyr^+^NF-κB p65^+^ fluorescence in *Cdc42* KO mice continuously treated with ZnO NPs (Fig. 6b) showed higher levels of Tyr^+^NF-κB p65^+^ staining after 14 days of treatment compared with day 0, with a further increase observed after 49 days of treatment.

To investigate whether NF-κB activation was accompanied by Bcl-2 expression, skin samples from *Cdc42* KO mice continuously treated with ZnO NPs were subjected to double immunofluorescence staining to identify NF-κB p65^+^ and Bcl-2^+^ cells (Fig. 6c). Few positive staining cells were observed in the skin of *Cdc42* KO mice at day 0. Image superimposition revealed that most Bcl-2^+^ cells detected in *Cdc42* KO mice after 14 and 49 days of continuous ZnO NP treatment were also NF-κB p65^+^. The quantitative analysis (Fig. 6d) showed higher numbers of NF-κB p65^+^Bcl-2^+^ cells in *Cdc42* KO mice after 14 days of continuous ZnO NP treatment compared with day 0, with a further increase in number after 49 days of treatment.

### ZnO NPs induce malignant transformation in melanocytes in vitro

**Fig. 7:**
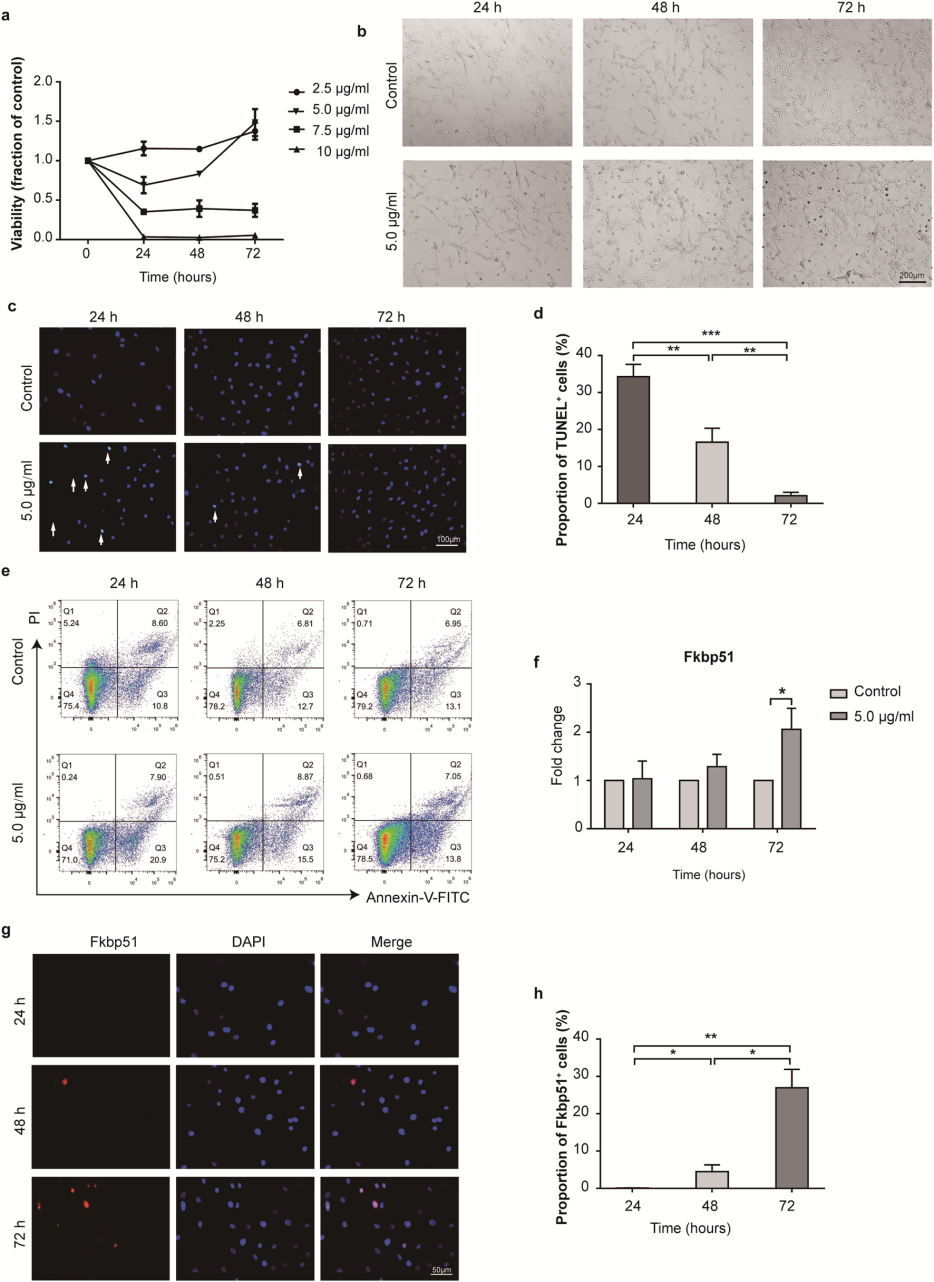
ZnO NP exposure induced malignant transformation in melanocytes. **a** The viability of HEMs treated with ZnO NPs for 24, 48, and 72 h after was measured using the CCK-8assay. The results are presented relative to the viability of untreated control cells. **b** Images of HEMs in culture after treatment with 5.0 µg/ml ZnO NPs, monitored using inverted phase-contrast microscopy. Scale bar = 200 µm. **c** TUNEL staining of HEMs after treatment with 5.0 µg/ml ZnO NPs. Scale bar = 100 µm. **d** Quantitation of TUNEL-stained cells after treatment with 5.0 µg/ml ZnO NPs. **e** Quantitative estimation of apoptosis induction was measured by Annexin-V/PI staining of HEMs after treatment with 5.0 µg/ml ZnO NPs for 24, 48 and 72 h. (Q4 represents normal cells; Q3 represents early apoptotic cells; and Q2 represents late apoptotic). **f** Fkbp51 mRNA levels were measured by qRT-PCR and normalized to *Gapdh* expression. **g** Immunofluorescence analysis of Fkbp51 in HEMs after treatment with 5.0 µg/ml ZnO NPs. Scale bar = 50 µm. **h** Quantitative analysis showing the proportion of Fkbp51^+^ cells in HEMs after treatment with 5.0 µg/ml ZnO NPs. Data represents Mean ± SD (n = 3). *p < 0.05, **p < 0.01 and ***p < 0.001

To further investigate the mechanism underlying the appearance of melanoma-like lesions in skin with epidermal barrier dysfunction following the infiltration of ZnO NPs, CCK-8 viability assay was performed to determine the safe concentration for ZnO NP exposure in HEMs (Fig. 7a). The viability was presented relative to the viability of untreated control cells. ZnO NPs at concentrations greater than 7.5 µg/ml reduced cell viability. Viability was approximately 35%, 39% and 37% in HEMs exposed to 7.5 µg/ml ZnO NPs after 24, 48, and 72 h. Exposure to a concentration of 10 µg/ml ZnO NPs resulted in a reduction in viability to approximately 3%, 3% and 5% after 24, 48, and 72 h. However, viability was approximately 69% and 83% after exposure to 5.0 µg/ml ZnO NPs for 24 and 48 h, increasing to approximately 148% after 72 h of exposure. Cells treated with 2.5 µg/ml ZnO NPs showed increased viability after 24, 48 and 72 h of exposure. These results were consistent with images of cell growth following treatment with ZnO NPs, which showed reduced cell numbers and the appearance of rounded cells detached from the growth surface after treatment with 5.0 µg/ml ZnO NPs (Fig. 7b). Cells treated with 2.5 µg/ml ZnO NPs (Additional file 1: Figure S5a) demonstrated consistent spread in a triangular shape on the growth surface, similar to untreated cells, indicating better cell survival compared with cells treated with 5.0 µg/ml ZnO NPs.

Apoptosis reflects the toxic effects of ZnO NPs on HEMs. Negligible TUNEL^+^ staining was observed in untreated control cells and cells treated with 2.5 µg/ml ZnO NPs (Additional file 1: Figure S5b). After treatment with 5.0 µg/ml ZnO NPs, a moderate number of TUNEL^+^ cells were observed after 24 h of treatment, a small number of TUNEL^+^ cells were observed after 48 h of treatment, and a minimal number of TUNEL^+^ cells were observed after 72 h of treatment (Fig. 7c). The quantitative analysis (Fig. 7d) revealed a gradually decreasing level of TUNEL^+^ staining with increased treatment time. Annexin-V/PI assay was used to distinguish apoptotic and necrotic cells. In Annexin-V/ PI analysis (Fig. 7e), 75.4%, 78.2% and 79.2% of HEMs remained viable in the control group after 24, 48 and 72 h. However, the apoptotic proportions of HEMs treated with 5.0 µg/ml ZnO NPs for 24, 48 and 72 h were 28.80% (including 20.9% of early apoptotic cells and 7.90% of late apoptotic cells), 24.37% (including 15.5% of early apoptotic cells and 8.87% of late apoptotic cells) and 20.85% (including 13.8% of early apoptotic cells and 7.05% of late apoptotic cells), respectively. Therefore, treatment with 5.0 µg/ml ZnO NPs for 72 h might induce an anti-apoptotic effect in melanocytes in vitro.

qRT-PCR was used to evaluate gene expression at the mRNA level. No changes in Fkbp51 mRNA levels in HEMs were observed after treatment with 2.5 µg/ml ZnO NPs compared with untreated cells (Additional file 1: Figure S5c). The Fkbp51 mRNA level in HEMs increased after treatment with 5.0 µg/ml ZnO NPs for 72 h compared with treatment for 24 and 48 h (Fig. 7f). Immunofluorescence analysis of Fkbp51 expression, was performed in HEMs after treatment with 5.0 µg/ml ZnO NPs (Fig. 7g). Few Fkbp51^+^ cells were observed in HEMs after treatment with 5.0 µg/ml ZnO NPs for 24 h, but some moderately Fkbp51^+^ positive cells were observed after treatment for 48 and 72 h. The quantitative analysis (Fig. 7h) revealed that 48 h of treatment with 5.0 µg/ml ZnO NPs in HEMS resulted in an increased proportion of Fkbp51^+^ cells compared with that observed after 24 h of treatment, and the proportion of Fkbp51^+^ cells increased further after 72 h of treatment.

These results indicate that treatment with 5.0 µg/ml ZnO NPs for 72 h might induce malignant transformation in melanocytes in vitro.

### ZnO NPs induces an anti-apoptotic effect in melanocytes through oxidative stress, mediated by the NF-κB pathway

**Fig. 8:**
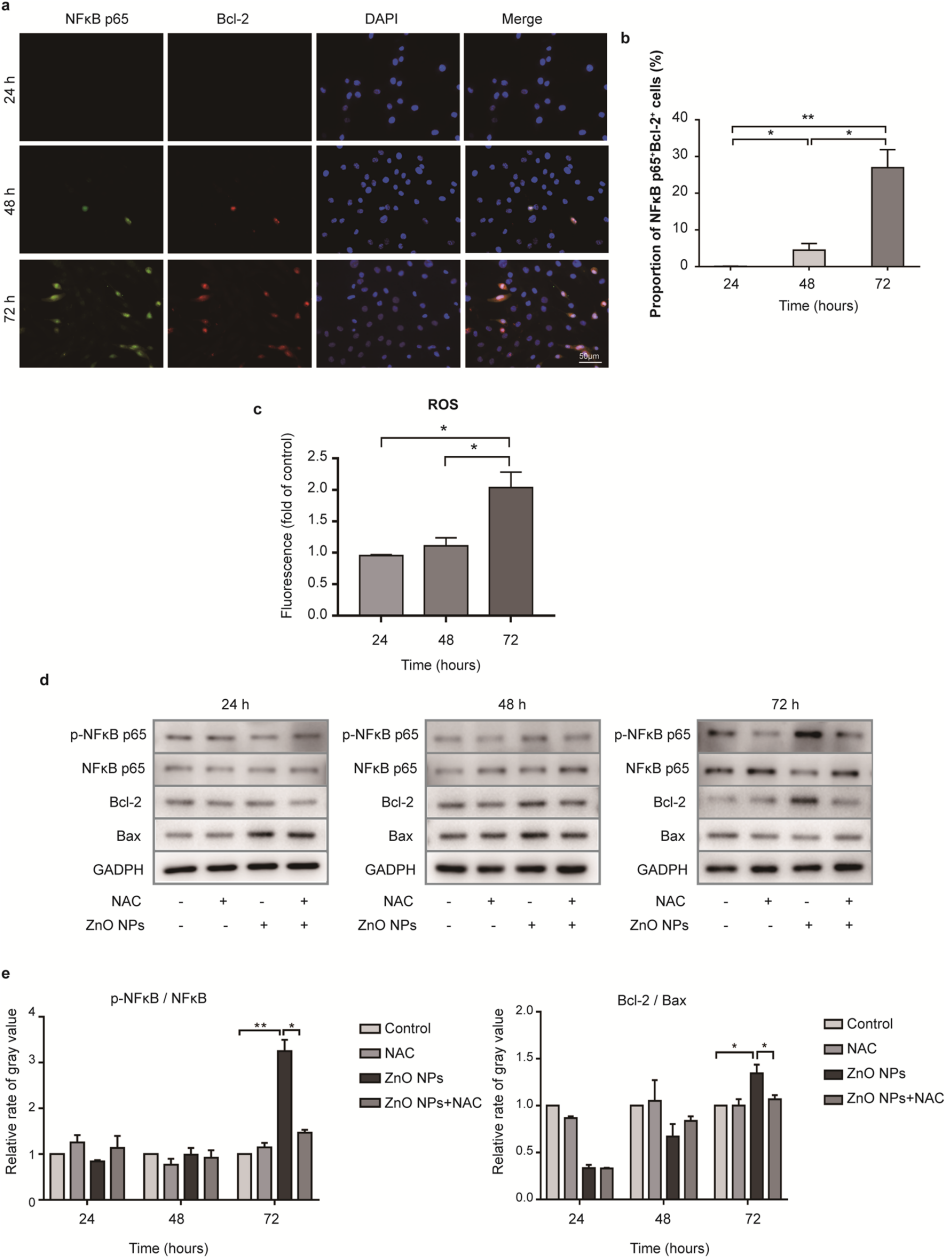
ZnO NPs induced an anti-apoptosis effect mediated by the activation of NF-κB in response to ROS. **a** Immunofluorescence analysis of skin sections using antibodies against NF-κB p65 and Bcl-2. Scale bar = 50 µm. **b** Quantitation of the proportion of NF-κB p65^+^Bcl-2^+^ cells after ZnO NP treatment. **c** Relative mean fluorescence intensity of intracellular ROS levels in HEMs treated with 5.0 µg/ml ZnO NPs compared with control levels. **d** Representative western blotting results showing the expression of NF-κB, p-NF-κB, Bcl-2, and Bax. n = 3. **e** Quantitation of ratio of p-NF-κB/NF-κB and Bcl-2/Bax. Data represents Mean ± SD (n = 3). *p < 0.05 and **p < 0.01

To investigate whether NF-κB activation was accompanied by Bcl-2 expression, HEMs were subjected to double immunofluorescence staining to identify NF-κB p65^+^ and Bcl-2^+^ cells after treatment with 5.0 µg/ml ZnO NPs for 24, 48, and 72 h (Fig. 8a). Few positively stained HEMs were detected after 24 h of treatment with 5.0 µg/ml ZnO NPs, but some moderately positive cells were detected after treatment for 48 and 72 h. Quantitative analysis of immunofluorescence after treatment with 5.0 µg/ml ZnO NPs (Fig. 8b) revealed that 48 h of treatment resulted in a higher proportion of NF-κB p65^+^Bcl-2^+^ HEM cells than 24 h of treatment, and the proportion of NF-κB p65^+^Bcl-2^+^ cells increased further after 72 h of treatment. This result indicated that treatment with 5.0 µg/ml ZnO NPs 72 h led to NF-κB activation, accompanied by Bcl-2 expression.

ZnO NPs have previously been shown to induce oxidative stress in treated cells. In our case, 5.0 µg/ml ZnO NP treatment for 24 and 48 h resulted in no changes in intracellular ROS content, whereas treatment for 72 h increased the ROS content in treated HEMs by approximately two fold that of the control cells (Fig. 8c).

NAC is a general inhibitor of ROS. To elucidate the role of ROS in mediating the malignant transformation of melanocytes, HEMs were treated with 5.0 µg/ml ZnO NPs with or without 2 h of 5 mM NAC pretreatment, followed by western blotting (Fig. 8d). After treatment with 5.0 µg/ml ZnO NPs for 72 h, the total cytoplasmic NF-κB level decreased, accompanied by an increase in cytoplasmic phosphorylated NF-κB, suggesting that NF-κB translocates into the nucleus, resulting in NF-κB activation. The expression of Bcl-2 increased after incubation with 5.0 µg/ml ZnO NPs for 72 h. As shown in Fig. 8e, the ratios of p-NF-κB/NF-κB and Bcl-2/Bax significantly increased in HEMs treated with 5.0 µg/ml ZnO NPs for 72 h, whereas the p-NF-κB/NF-κB and Bcl-2/Bax ratios were significantly decreased in cells pretreated with NAC. These results suggested that ZnO NPs induce intracellular ROS, which trigger an anti-apoptosis effect mediated by NF-κB activation.

## Discussion

Sunscreens are widely used consumer products that may contain nanosized, inorganic, UV-filtering agents, such as ZnO. The safety of ZnO NPs used for topical application has been evaluated using both in vitro and in vivo models, but the results emerging from various studies are inconclusive and controversial. Due to the increasing prevalence of diseases associated with epidermal barrier dysfunction, such as atopic dermatitis, eczema, and acne, whether the repetitive application of ZnO NPs to damaged skin is associated with an increased risk of penetration into the deep skin layers must be determined [1, 12]. In our study, we used a mouse model of epidermal barrier dysfunction, in which skin damage was caused by the skin keratinocyte-specific deletion of *Cdc42* [11].

To obtain a dispersion pattern and consistency similar to those found in sunscreens, ZnO mixtures were prepared in this study using the same ZnO NP content typically allowed in commercial products at a dose of 2 mg/cm^2^ [28]. In this study, we used 50 nm ZnO NPs, which were used in previous studies [12]. ZnO NPs were detected in the stratum corneum and the basal layer of the epidermis when applied to skin with epidermal barrier dysfunction after 14 days of treatment, which indicated that skin with epidermal barrier dysfunction might experience the penetration and translocation of ZnO NPs following repeated applications. ZnO NPs also reached the stratum basale of the epidermis, where melanocytes are located. This study aimed to investigate the effects of the penetrating ZnO NPs on melanocytes in an epidermal barrier dysfunction model mouse.

Melanocytes are located in the basal layer of the epidermis, where they maintain a life-long and stable ratio with basal keratinocytes. Melanocytes represent a minority cell population within the basal epidermis and divide infrequently [40, 41]. Cdc42 deficiency results in the loss of intercellular junctions in the epidermis [11]. which may allow melanocytes to escape keratinocyte control, promoting abnormal proliferation. The proliferation of melanocytes with enlarged nuclei that grow in an irregular pattern within the epidermis could lead to melanoma in situ. Once melanoma cells leave the epidermis and enter the dermis, melanocytes undergo a malignant transformation and acquire the characteristics of invasive cells [42]. The present study revealed that ZnO NP exposure induced a significantly pigmented appearance in epidermal barrier dysfunction model mice after 14 and 49 days of continuous ZnO NP treatment, and hyperpigmentation was further supported by a markedly increased number of melanocytes in the epidermis and dermis after 14 and 49 days of treatment (Tyr/Dct staining). In our study, increased cells with large and irregular nuclei were found in the hyperplastic epidermis in epidermal barrier dysfunction model mice continuously treated with ZnO NPs after 14 and 49 days, and cells with obvious melanin granules were found in a deeper layer of the dermis in epidermal barrier dysfunction model mice treated continuously with ZnO NPs after 49 days. The melanin granules found in a deeper layer of the dermis were accompanied with local immune microenvironment changed, 49-day ZnO NPs treatment resulted in the recruitment of DC cells in the dermis of epidermal barrier dysfunction skin. NP treatment has been hypothesized to cause the transformation of normal cells into tumor cells [15, 43] We further investigated whether abnormal proliferation and activation can lead to the malignant transformation of melanocytes after ZnO NP treatment.

In our study, RNA-seq was further used to provide substantial and detailed information regarding the toxicological responses associated with malignant transformation, and results of the qRT-PCR analysis revealed comparable results to those obtained by RNA-seq. FKBP51 has been found to be highly expressed in melanoma, which may be associated with a melanoma cancer stem-cell phenotype and melanoma invasiveness [44, 45]. In addition, Rambow et al. suggested that TRIM63 was overexpressed in melanoma cell lines [46]. In our study, the levels of both *Fkbp51* and *Trim63* were clearly upregulated after 14 days of ZnO NP treatment to the exposed skin of epidermal barrier dysfunction model mice. TSP1 expression is also thought to suppresses angiogenesis in melanoma [47]. We also found that *Tsp1* mRNA levels were suppressed by 14 days of ZnO NP treatment applied to the skin of epidermal barrier dysfunction model mice. These results indicated that continuous ZnO NP treatment for 14 days induced the expression of melanoma-related genes in the epidermal barrier dysfunction model mouse. Double immunofluorescence staining for Dct and Fkbp51 showed that most of the Dct^+^ cells were also Fkbp51^+^ in the skin of epidermal barrier dysfunction model mice continuously treated with ZnO NPs after 14 and 49 days. These results indicate that the continuous application of ZnO NPs to skin with epidermal barrier dysfunction resulted in a persistent increase in Fkbp51 expression in melanocytes. Thus, continuous and repeated exposure (for 14 days or longer) to ZnO NPs induced a malignant transformation in melanocytes, resulting in the development of melanoma-like lesions in epidermal barrier dysfunction model mice in vivo.

Oxidative stress is an important mechanism implicated in NP-induced effects on health, such as cancer development [48–50]. It has been suggested that NPs led to changes of oxidative stress indicators like CAT, SOD, GSH and MDA [51]. In our study, oxidative stress was found to be induced by the application of ZnO NPs to the skin of epidermal barrier dysfunction model mice, as evidenced by GSH depletion, reductions in CAT and SOD activity, and increased MDA content. An increase in the level of 8-OHdG staining in the skin of epidermal barrier dysfunction model mice after continuous ZnO NP treatment revealed DNA damage associated with oxidative stress. Epidermal melanocytes are particularly vulnerable to oxidative stress due to the pro-oxidant state generated during melanin synthesis but intrinsic antioxidant defense generated under pathologic conditions [20]. Recent observations have suggested that melanomagenesis may be associated with increased ROS, oxidative stress, and redox imbalance [52–57]. The redox status has been suggested to play an important role in melanocyte transformation [27], and increased ROS levels accelerate melanoma initiation and progression [58]. It has been suggested that increased ROS fluorescence was observed after exposure to the ZnO nanomaterials [59]. In our study, HEMs treated with 5.0 µg/ml ZnO NPs for 72 h presented with significantly increased ROS content, whereas the same treatment for 24 and 48 h had no significant effect on intracellular ROS content compared with untreated control.

The ROS-generating capability of ZnO NPs is associated with the cytotoxic response, which could lead to cell death or apoptosis [59–62]. The deregulation of apoptosis is considered a hallmark feature of cancer [63, 64]. In our study, increased TUNEL staining was observed following 4 days of continuous ZnO NP treatment applied to the skin of epidermal barrier dysfunction model mice; however, TUNEL^+^ cells decreased after 14 and 49 days of continuous treatment, suggesting that continuous ZnO NP treatment decreased apoptosis in the skin of epidermal barrier dysfunction model mice. Consistently, in vitro, a moderate number of TUNEL^+^ cells were observed after 24 h of treatment with 5.0 µg/ml ZnO NPs, but a decrease in the number of TUNEL^+^ cells was detected in HEMs after 48 and 72 h of treatment. Annexin-V/PI assay showed a decreased proportions of apoptotic HEMs treated with 5.0 µg/ml ZnO NPs for 72 h than 24 and 48 h as well. The Bcl-2 family of proteins, which are closely associated with cancer development, are key players in apoptosis, with some members, including Bcl-2 and Bcl-x_L_, acting as apoptosis inhibitory proteins and others, such as Bax, Bad, and Bak, serving as apoptosis promoters [65–67]. In our experiment, we found that melanocytes were the primary source of Bcl-2 production in skin with epidermal barrier dysfunction, as indicated by the increased intensity of Dct^+^Bcl-2^+^ staining after continuous ZnO NP treatment for 14 and 49 days. Thus, our results revealed that continuous exposure to ZnO NPs deregulated apoptosis and upregulated the expression of the anti-apoptotic factor Bcl-2 in melanocytes.

Increased Bcl-2 protein expression has been associated with NF-κB/Rel activation [40, 68]. resulting in the nuclear translocation of NF-κB p65. The results of our study showed that melanocytes were the primary source of NF-κB p65 activation. Further study revealed that increased NF-κB p65 expression was accompanied by an increase in Bcl-2 expression in the skin of epidermal barrier dysfunction model mice after continuous ZnO NP treatment for 14 and 49 days. NF-κB activation and upregulated Bcl-2 expression were also observed in HEMs after treatment with 5.0 µg/ml ZnO NPs for 72 h in vitro. Our study revealed that continuous exposure to ZnO NPs promoted an anti-apoptotic effect in melanocytes, mediated by NF-κB signaling.

**Fig. 9:**
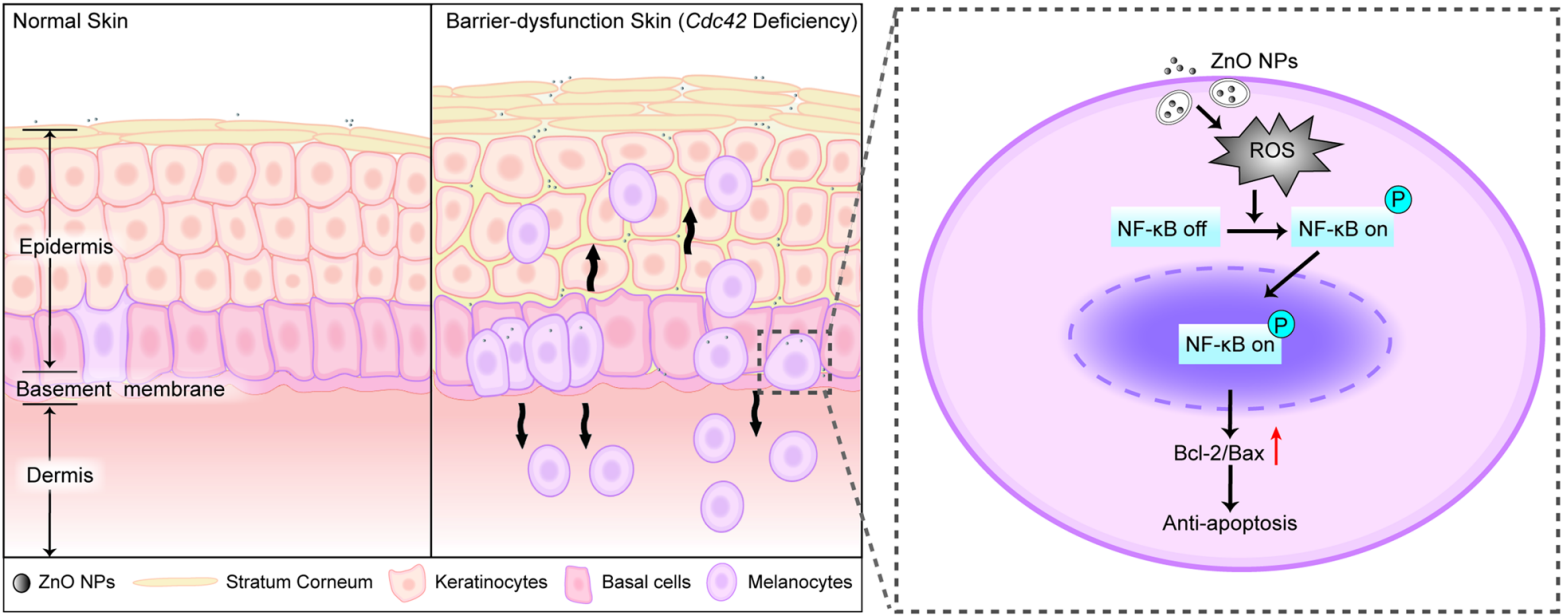
A schematic illustration showing the impact of ZnO NPs on skin with barrier dysfunction. In normal skin, ZnO NPs cannot penetrate the stratum corneum. In skin with barrier dysfunction, ZnO NPs are able to penetrate the stratum basale of the epidermis, resulting in melanocyte hyperplasia in the epidermis and dermis. Moreover, ZnO NPs induce an anti-apoptotic effect in melanocytes via the activation of the NF-κB pathway, which is mediated by oxidative stress

Intracellular oxidative stress can induce NF-κB activation and alter the activity of other transcription factors, triggering a downstream stress response, including an anti-apoptotic effect, during the malignant transformation of melanocytes [68–70]. The antioxidant agent NAC was used to investigate whether continuous exposure to ZnO NPs and the associated upregulation of ROS induced NF-κB activation to promote anti-apoptotic factors in vitro. The results of our study demonstrated that ROS induction following continuous ZnO NP exposure led to NF-κB activation and an increase in the Bcl-2/Bax ratio. NAC pretreatment efficiently inhibited the induction of an apoptotic effect by ZnO NPs (as measured by the Bcl-2/Bax ratio) and resulted in reduced nuclear NF-κB p65 expression (as measured by the p-NF-κB/NF-κB ratio). Thus, ZnO NPs appear to induce a high level of ROS in melanocytes, triggering an anti-apoptotic effect mediated by the activation of the NF-κB pathway. The penetration of ZnO NPs into the skin under conditions of epidermal barrier dysfunction and the resultant damage to melanocytes are depicted in Fig. 9.

## Conclusion

Our results demonstrate that repeated and continuous topical exposure to ZnO NPs in skin with barrier dysfunction allows these particles to penetrate into the stratum basale of the epidermis, resulting in the development of melanoma-like lesions on the skin in a mouse model of epidermal barrier dysfunction. Continuous exposure to ZnO NPs induces an anti-apoptotic effect in melanocytes through the oxidative stress–mediated activation of NF-κB pathways, both in vivo and in vitro. This study provided new information regarding the melanoma risk associated with repeated and continuous exposure to ZnO NPs under conditions of epidermal barrier dysfunction.

## Supplementary Information

**Additional file 1: Figure S1**. PCR verification of *Cdc42* deletion in the epidermis. The upper panel shows PCR products for the *Cdc42*^*loxp/loxp*^ allele and the bottom panel shows those of the K5-Cre (+) allele. The 700-base-pair (bp) band represents the flox allele; the 600 bp band represents the wild-type (WT) allele; the 667 bp band represents the K5-Cre (+) allele; and the blank band represents the internal positive control. The *Cdc42*^*loxp/loxp*^/Cre+ mice were *Cdc42* KO, and the* Cdc42*^*loxp/wt*^/Cre-/*Cdc42*^*loxp/loxp*^/Cre- were *Cdc42* WT. **Figure S2**. Cluster Analysis Using a Heatmap. Cluster analysis of differentially expressed mRNA in the skin of WT and *Cdc42* KO mice continuously treated for 14 days in the negative control and zinc oxide nanoparticles (ZnO NPs) group. Red indicates increased expression, and blue indicates decreased expression. n = 3. **Figure S3**. Changes in skin of WT and *Cdc42* KO mice continuously treated in the negative control group. a The gross morphology of WT and *Cdc42* KO mice in the negative control group continuously treated for 0, 14 and 49 days. b Histological changes in the skin of WT and *Cdc42* KO mice in the negative control group continuously treated for 0, 14 and 49 days. Scale bar = 100 μm. c Immunohistochemical detection of tyrosinase in the skin of WT and *Cdc42* KO mice in the negative control group continuously treated for 0, 14 and 49 days. Scale bar = 50 µm. **Figure S4**. Abnormal apoptosis and proliferation in the skin of WT and *Cdc42* KO mice treated with ZnO NPs. a Terminal deoxynucleotidyl transferase dUTP nick end labeling (TUNEL) in the skin of WT and *Cdc42* KO mice continuously treated with ZnO NPs, observed on days 4. Scale bar = 100 μm. b Quantitation of TUNEL-positive cells in the epidermis of *Cdc42* KO continuously treated with ZnO NPs for 4, 14 and 49 days. c Immunohistochemical image of Ki67-positive staining cells in the epidermis of WT and *Cdc42* KO mice continuously treated with ZnO NPs, observed on days 0, 14 and 49. Scale bar = 50 μm. d Quantitative analysis of the number of Ki67-positive cells in the epidermis of WT and *Cdc42* KO mice continuously treated with ZnO NPs for 4, 14, and 49 days. Data represents Mean ± standard deviation (SD) (n=3). *p < 0.05, **p < 0.01, and ***p < 0.001. **Figure S5**. Changes in human epidermal melanocytes (HEMs) in culture after treatment with 2.5 µg/ml ZnO NPs. a Images of HEMs in culture after treatment with 2.5 µg/ml ZnO NPs, monitored using inverted phase-contrast microscopy. Scale bar = 200 μm. b TUNEL staining of HEMs after treatment with 2.5 µg/ml ZnO NPs. Scale bar = 100 µm. c Fkbp51 mRNA levels were measured by quantitative real-time reverse transcriptase PCR (qRT-PCR) and normalized to Gapdh expression. Data represents Mean ± SD (n = 3).

## Data Availability

The datasets used and/or analyzed in the current study are available from the corresponding author on reasonable request.

## Abbreviations

AD: Atopic dermatitis
CAT: Catalase
Cdc42: Cell division cycle 42;
CCK-8: Cell-counting kit 8
DAPI: 4’,6-Diamidino-2-phenylindole
DCFH-DA: 2′,7′ -Dichlorofluorescein diacetate
DE: Differentially expressed
Fkbp51: FK506-binding protein 51
GSH: Glutathione
HPMC: Hydroxypropyl methylcellulose
HEMs: Human epidermal melanocytes
KO: Knockout
MDA: Malondialdehyde
NAC: N-acetylcysteine
NS: Normal saline
NF-κB: Nuclear factor kappa B
PBS: Phosphate-buffered saline
P-NF-κB: Phospho-NF-κB
QRT-PCR: Quantitative real-time reverse transcriptase PCR
ROS: Reactive oxygen species
RNA-seq: RNA sequencing
SEM: Scanning electron microscopy
SD: Standard deviation
TAM: Tumor-associated macrophages
TEM: Transmission electron microscopy
TEWL: Transepidermal water loss
Treg: Regulatory T cells
TUNEL: Terminal deoxynucleotidyl transferase dUTP nick end labeling
Tsp1: Thrombospondin-1
T-SOD: Total superoxide dismutase
Trim63: Tripartite motif-containing 63
TME: Tumor microenvironment
Tyr: Tyrosinase
Trp-2/Dct: Tyrosinase-related protein-2/dopachrome tautomerase
UV: Ultraviolet
WT: Wild-type
XRD: X-ray diffraction
ZnO NPs: Zinc oxide nanoparticles
8-OHdG: 8-Hydroxydeoxyguanosine
