# Supplementary material for: Continuous ZnO nanoparticle exposure induces melanoma-like skin lesions in epidermal barrier dysfunction model mice through anti-apoptotic effects mediated by the oxidative stress–activated NF-κB pathway
Source: J Nanobiotechnology. 2022 Mar 5;20:111. doi: 10.1186/s12951-022-01308-w (PMC8898538; doi:10.1186/s12951-022-01308-w)
Supplement: Supplementary file 1 — Additional file 1: Figure S1. PCR verification of Cdc42 deletion in the epidermis. The upper panel shows PCR products for the Cdc42loxp/loxp allele and the bottom panel shows those of the K5-Cre (+) allele. The 700-base-pair (bp) band represents the flox allele; the 600 bp band represents the wild-type (WT) allele; the 667 bp band represents the K5-Cre (+) allele; and the blank band represents the internal positive control. The Cdc42loxp/loxp/Cre+ mice were Cdc42 KO, and the Cdc42loxp/wt/Cre-/Cdc42loxp/loxp/Cre- were Cdc42 WT. Figure S2. Cluster Analysis Using a Heatmap. Cluster analysis of differentially expressed mRNA in the skin of WT and Cdc42 KO mice continuously treated for 14 days in the negative control and zinc oxide nanoparticles (ZnO NPs) group. Red indicates increased expression, and blue indicates decreased expression. n = 3. Figure S3. Changes in skin of WT and Cdc42 KO mice continuously treated in the negative control group. a The gross morphology of WT and Cdc42 KO mice in the negative control group continuously treated for 0, 14 and 49 days. b Histological changes in the skin of WT and Cdc42 KO mice in the negative control group continuously treated for 0, 14 and 49 days. Scale bar = 100 μm. c Immunohistochemical detection of tyrosinase in the skin of WT and Cdc42 KO mice in the negative control group continuously treated for 0, 14 and 49 days. Scale bar = 50 µm. Figure S4. Abnormal apoptosis and proliferation in the skin of WT and Cdc42 KO mice treated with ZnO NPs. a Terminal deoxynucleotidyl transferase dUTP nick end labeling (TUNEL) in the skin of WT and Cdc42 KO mice continuously treated with ZnO NPs, observed on days 4. Scale bar = 100 μm. b Quantitation of TUNEL-positive cells in the epidermis of Cdc42 KO continuously treated with ZnO NPs for 4, 14 and 49 days. c Immunohistochemical image of Ki67-positive staining cells in the epidermis of WT and Cdc42 KO mice continuously treated with ZnO NPs, observed on days 0, 14 and 49. Scale bar = 50 μm [file 12951_2022_1308_MOESM1_ESM.doc]

**Additional File**

Continuous ZnO nanoparticle exposure induces melanoma-like skin lesions in epidermal barrier dysfunction model mice through anti-apoptotic effects mediated by the oxidative stress–activated NF-κB pathway

*Ping Wang^1,†^, Guodong Hu^2,†^, Wen Zhao^3^, Juan Du^4^, Menghan You^1^, Mengying Xv^5^, Hong Yang^2^, Min Zhang^1^, Fang Yan^1^, Mianbo Huang^1^, Xueer Wang^1^, Lin Zhang^1^,*, Yinghua Chen^1^,**

**1. Additional Figures**

**1.1. *Cdc42* knockout mice show abnormal inside-out and outside-in barrier function.**


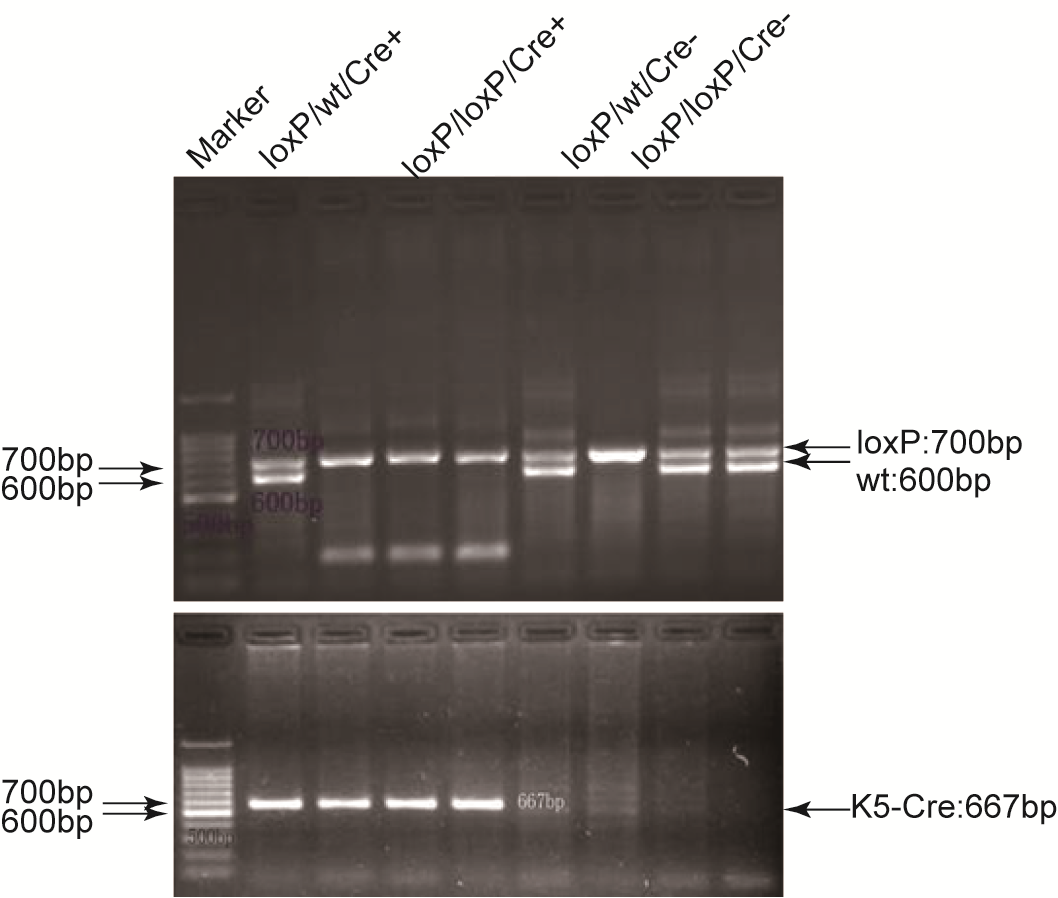


**Figure S1.**  PCR verification of *Cdc42* deletion in the epidermis. The upper panel shows PCR products for the *Cdc42*^loxp/loxp^ allele and the bottom panel shows those of the *K5*-Cre (+) allele. The 700-base-pair (bp) band represents the flox allele; the 600 bp band represents the wild-type (WT) allele; the 667 bp band represents the *K5*-Cre (+) allele; and the blank band represents the internal positive control. The *Cdc42*^loxp/loxp/Cre+^ mice were *Cdc42* KO, and the *Cdc42*^loxp/wt/Cre-^/*Cdc42*^loxp/loxp/Cre-^ were *Cdc42* WT.

**1.2. [In](../../../luckypp/AppData/Local/youdao/dict/Application/8.9.6.0/resultui/html/index.html#/javascript:;) [vivo](../../../luckypp/AppData/Local/youdao/dict/Application/8.9.6.0/resultui/html/index.html#/javascript:;) [experiment](../../../luckypp/AppData/Local/youdao/dict/Application/8.9.6.0/resultui/html/index.html#/javascript:;)s**

**
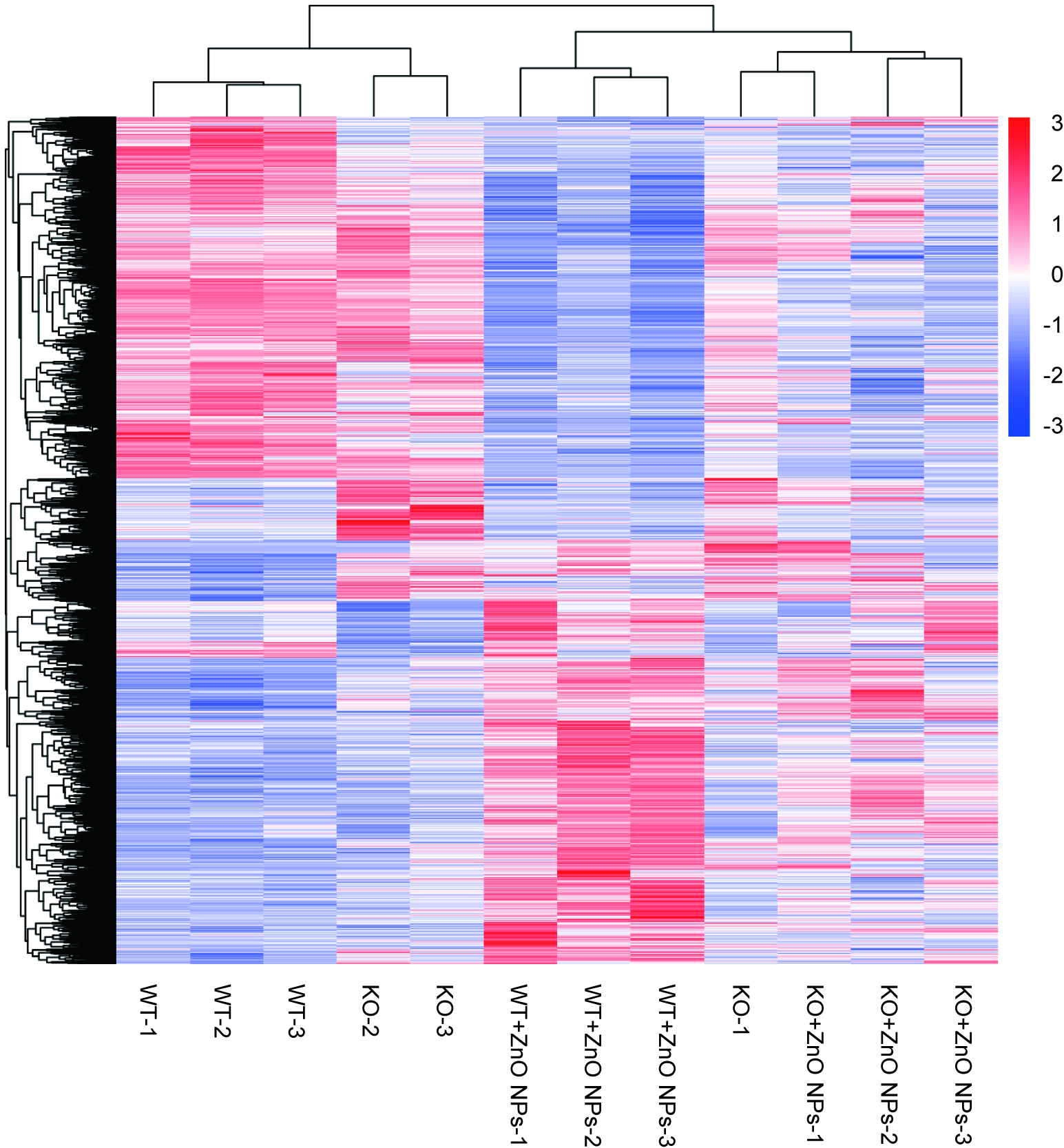
**

**Figure S2.** Cluster Analysis Using a Heatmap. Cluster analysis of differentially expressed mRNA in the skin of WT and *Cdc42* KO mice continuously treated for 14 days in the negative control and zinc oxide nanoparticles (ZnO NPs) group. Red indicates increased expression, and blue indicates decreased expression. n=3.

**
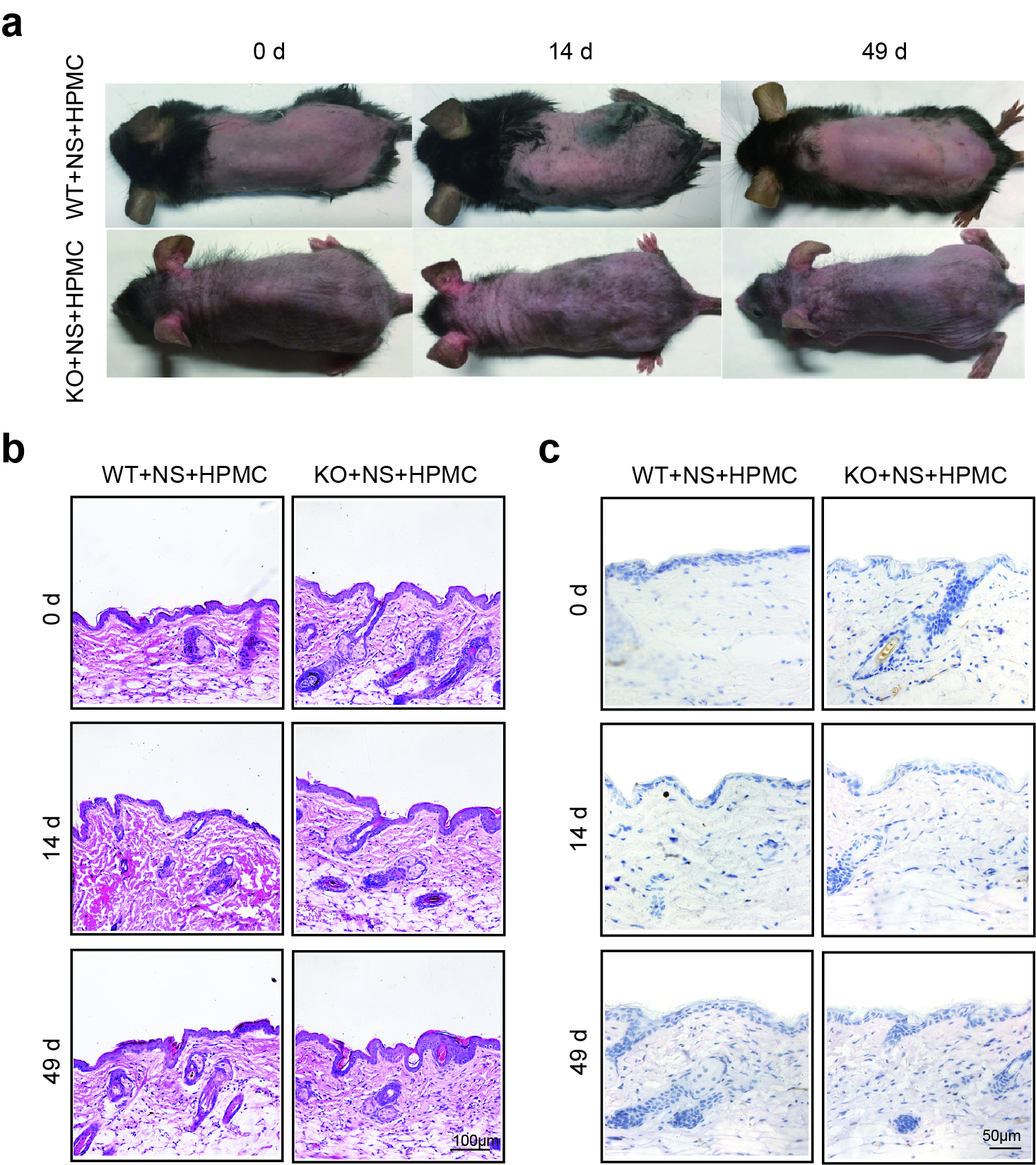
**

**Figure S3.** Changes in skin of WT and *Cdc42* KO mice continuously treated in the negative control group. **a** The gross morphology of WT and *Cdc42* KO mice in the negative control group continuously treated for 0, 14 and 49 days. **b** Histological changes in the skin of WT and *Cdc42* KO mice in the negative control group continuously treated for 0, 14 and 49 days. Scale bar = 100 μm. **c** Immunohistochemical detection of tyrosinase in the skin of WT and *Cdc42* KO mice in the negative control group continuously treated for 0, 14 and 49 days. Scale bar = 50 μm.


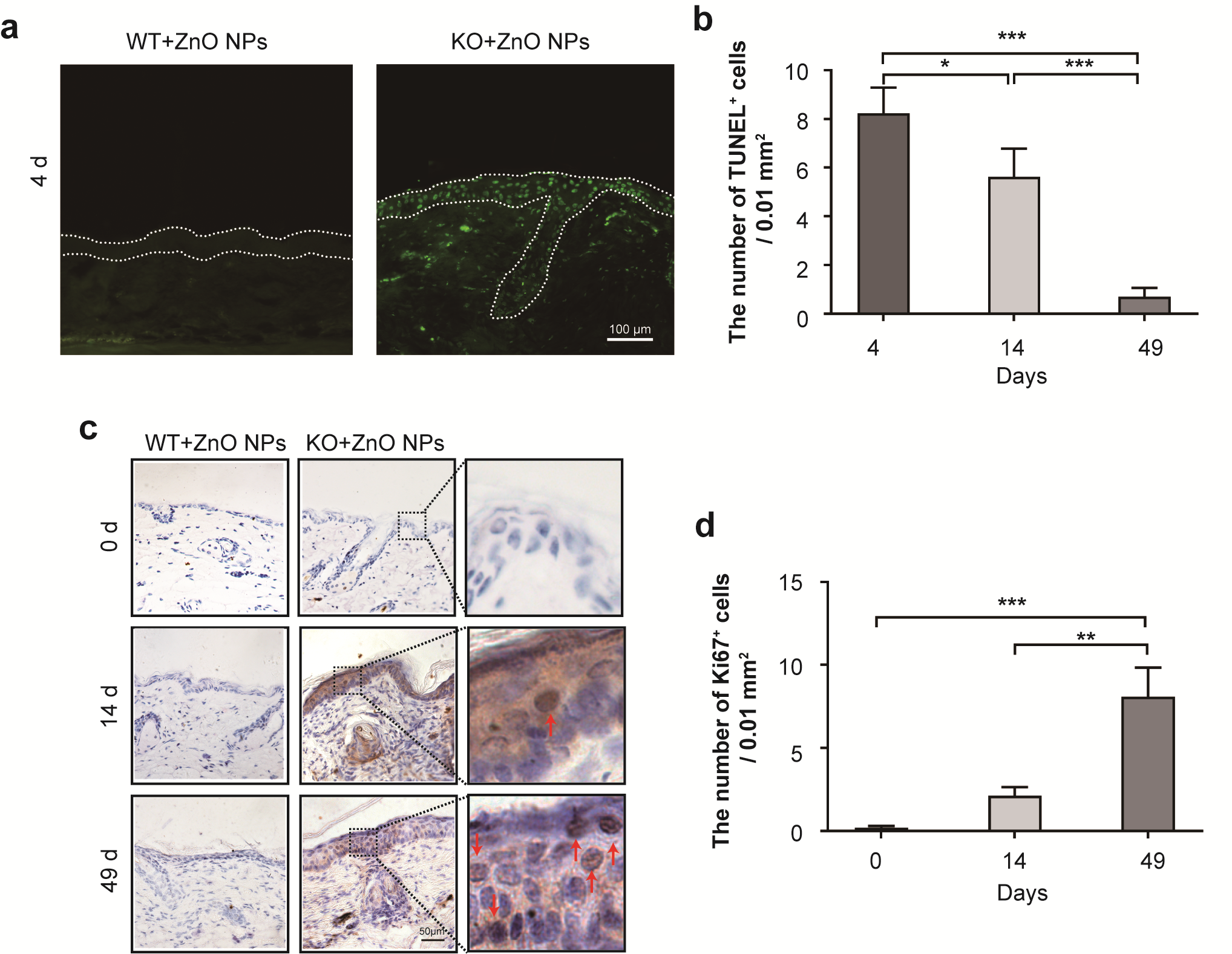


**Figure S4.** Abnormal apoptosis and proliferation in the skin of WT and *Cdc42* KO mice treated with ZnO NPs. **a** Terminal deoxynucleotidyl transferase dUTP nick end labeling (TUNEL) in the skin of WT and *Cdc42* KO mice continuously treated with ZnO NPs, observed on days 4. Scale bar = 100 μm. **b** Quantitation of TUNEL-positive cells in the epidermis of *Cdc42* KO continuously treated with ZnO NPs for 4, 14 and 49 days. **c** Immunohistochemical image of Ki67-positive staining cells in the epidermis of WT and *Cdc42* KO mice continuously treated with ZnO NPs, observed on days 0, 14 and 49. Scale bar = 50 μm. **d** Quantitative analysis of the number of Ki67-positive cells in the epidermis of WT and *Cdc42* KO mice continuously treated with ZnO NPs for ~~4~~, 14, and 49 days. Data represents Mean ± standard deviation (SD) (n=3). *p < 0.05, **p＜0.01, and ***p＜0.001.

**2.3. [In](../../../luckypp/AppData/Local/youdao/dict/Application/8.9.6.0/resultui/html/index.html#/javascript:;) [vitro](../../../luckypp/AppData/Local/youdao/dict/Application/8.9.6.0/resultui/html/index.html#/javascript:;) [experiment](../../../luckypp/AppData/Local/youdao/dict/Application/8.9.6.0/resultui/html/index.html#/javascript:;)s**


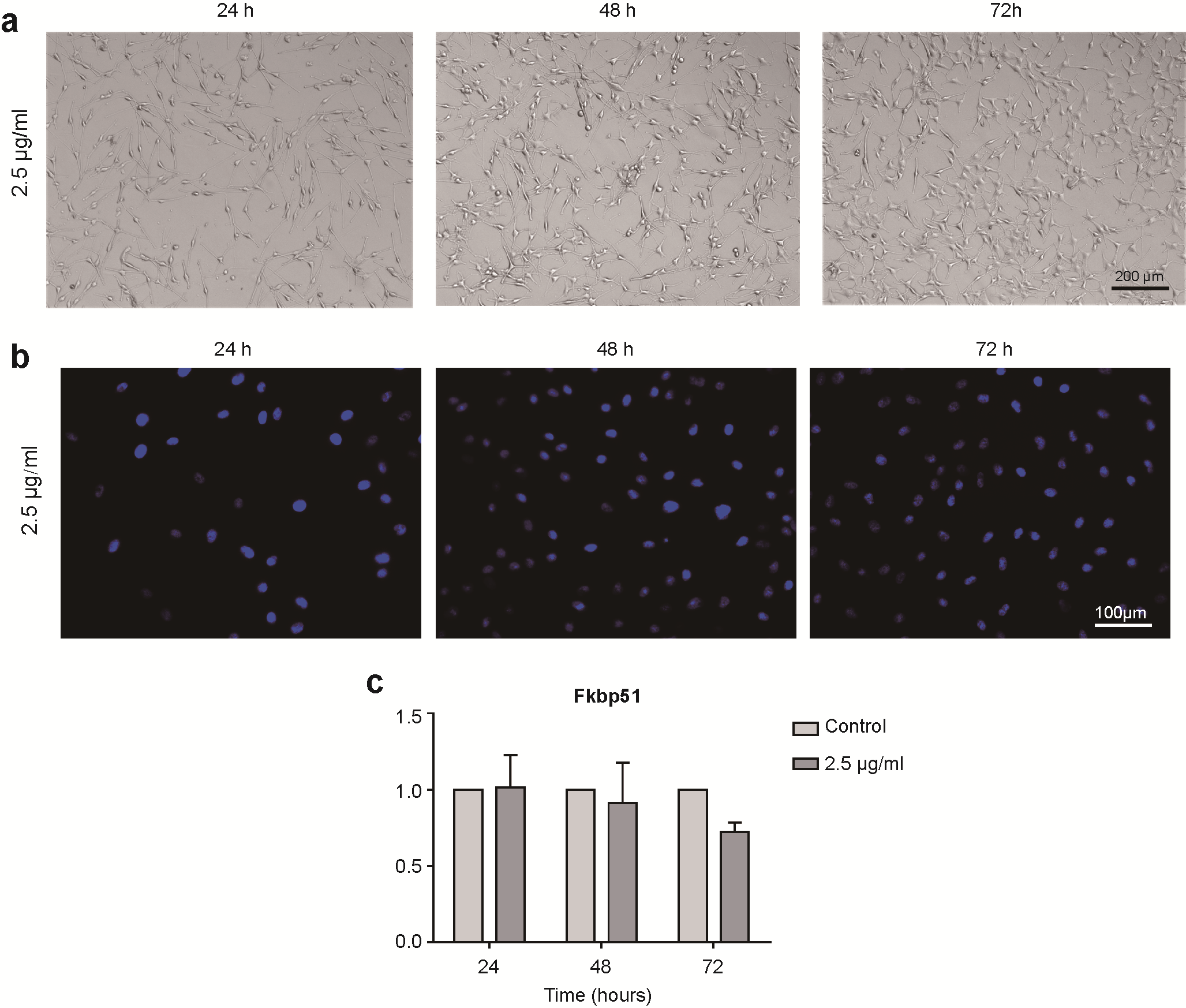


**Figure S5.** Changes in human epidermal melanocytes (HEMs) in culture after treatment with 2.5 µg/ml ZnO NPs. **a** Images of HEMs in culture after treatment with 2.5 µg/ml ZnO NPs, monitored using inverted phase-contrast microscopy. Scale bar = 200 µm. **b** TUNEL staining of HEMs after treatment with 2.5 µg/ml ZnO NPs. Scale bar = 100 µm. **c** Fkbp51 mRNA levels were measured by quantitative real-time reverse transcriptase PCR (qRT-PCR) and normalized to *Gapdh* expression. Data represents Mean ± SD (n = 3).
